# Structural and functional gastrointestinal abnormalities in ACTA2 R179H mice modeling multisystemic smooth muscle dysfunction syndrome

**DOI:** 10.1172/jci.insight.190469

**Published:** 2026-01-06

**Authors:** Ahmed A. Rahman, Rhian Stavely, Leah C. Ott, Christopher Y. Han, Kensuke Ohishi, Ryo Hotta, Alan J. Burns, Sabyasachi Das, Emily Da Cruz, Diana Tambala, Mark E. Lindsay, Patricia L. Musolino, Allan M. Goldstein

**Affiliations:** 1 Department of Pediatric Surgery, Massachusetts General Hospital, Harvard Medical School, Boston, Massachusetts, USA.; 2 Drug Discovery Laboratory, Wakunaga Pharmaceutical Co., Ltd., Akitakata, Hiroshima, Japan.; 3 Center for Genomic Medicine, Massachusetts General Hospital, Boston, Massachusetts, USA.; 4 Department of Neurology, Massachusetts General Hospital, Harvard Medical School, Boston, Massachusetts, USA.; 5 Cardiovascular Genetics Program;; 6 Cardiovascular Research Center, Division of Cardiology, Department of Medicine; and; 7 Division of Neurocritical Care, Department of Neurology, Massachusetts General Hospital, Boston, Massachusetts, USA.

**Keywords:** Gastroenterology, Neuroscience, Genetic diseases

## Abstract

Multisystemic smooth muscle dysfunction syndrome (MSMDS) is a rare disorder caused by ACTA2 mutations, including the R179H variant, which alters actin filament stability and dynamics and smooth muscle contractility. Cardiovascular complications dominate its clinical presentation, but gastrointestinal (GI) dysfunction significantly affects quality of life. To investigate the structural, functional, and cellular basis of gut dysmotility in MSMDS, we reviewed clinical data from 24 patients with MSMDS and studied the ACTA2 R179H mouse model. Patients exhibited severe gut dysmotility, with 75% requiring medication for chronic constipation. ACTA2 mutant mice displayed cecal and colonic dilatation, reduced intestinal length, and disrupted colonic migrating motor complexes. Delayed whole-gut transit and impaired contractile responses to electrical and pharmacological stimulation were observed. Transcriptomic analysis revealed significant actin cytoskeleton-related gene changes in smooth muscle cells, and immune profiling identified increased lymphocytic infiltration. Despite functional abnormalities, there were no obvious changes in the enteric nervous system. These findings establish ACTA2 mice as a robust model for studying GI pathology in MSMDS, elucidating the role of smooth muscle dysfunction in gut dysmotility. This model provides a foundation for developing targeted therapies aimed at restoring intestinal motility by directly addressing actin cytoskeletal disruptions in smooth muscle cells.

## Abstract 2

ACTA2 mutation-driven intestinal dysmotility is linked to disrupted smooth muscle function, highlighting pathways for therapeutic strategies to restore motility in multisystemic smooth muscle dysfunction syndrome.

## Graphical abstract

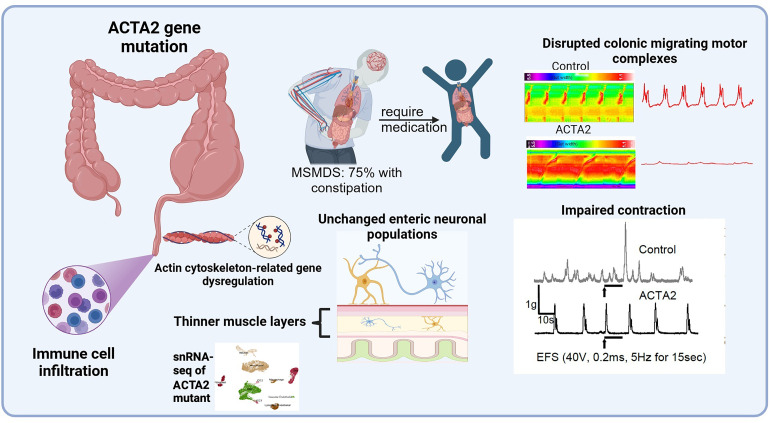

## Introduction

Multisystemic smooth muscle dysfunction syndrome (MSMDS) is a rare, life-threatening pediatric condition characterized by profound multisystemic impairments due to mutations in the *ACTA2* gene encoding smooth muscle α-actin (α-SMA), which is expressed in both vascular and visceral smooth muscle cells. The most severe *ACTA2* mutations involve replacement of arginine 179, most frequently with histidine (R179H), although R179C and R179S have also been observed. These substitutions disrupt actin polymerization, impairing smooth muscle contractility (1–3). Clinically, manifestations of MSMDS include congenital mydriasis, patent ductus arteriosus, cerebrovascular disease, aortic aneurysms, hypotonic bladder, and intestinal dysmotility, all of which are due to smooth muscle dysfunction in the vasculature and in the visceral organs (4–10).

Smooth muscle cells are essential for normal function of the gastrointestinal (GI) tract, contributing to gut motility and the regulation of luminal pressure. Through rhythmic contraction and relaxation, smooth muscle cells generate peristaltic waves that propel luminal contents and facilitate effective digestion and elimination of waste. These cells are integral components of the so-called SIP syncytium, a functional network composed of smooth muscle cells, interstitial cells of Cajal (ICCs), and PDGFRα^+^ cells. The interplay between these cell types, along with the enteric nervous system (ENS), orchestrates the electrical and mechanical activities of the gut to sustain coordinated motility (11–17).

Disruption in the function of any component of the SIP syncytium, particularly smooth muscle cells, can severely affect gut motility. In the context of MSMDS, mutations in the *ACTA2* gene lead to structural and functional abnormalities in smooth muscle cells, resulting in impaired contractility (2, 8). Because SIP syncytium components are functionally interconnected, smooth muscle cell dysfunction likely influences neighboring ICCs and PDGFRα^+^ cells through altered mechanical and paracrine signaling, thereby contributing to dysregulation of gut motility. The resulting dysmotility can lead to a variety of GI manifestations, including delayed gastric emptying, intestinal pseudo-obstruction, intractable constipation, abdominal pain, distension, and a number of other symptoms that compromise quality of life for affected individuals (18–20).

Advancements in genomic technologies, particularly exome sequencing, have enhanced the early diagnosis of MSMDS, creating opportunities for timely intervention before irreversible damage sets in. However, no disease-modifying treatments are available to mitigate the neurological, vascular, or GI consequences of the disease (21–23). This underscores the urgent need to understand the mechanisms underlying MSMDS, especially in the context of smooth muscle dysfunction, which plays a pivotal role in both vascular and GI systems. The development of a robust animal model of MSMDS has been a significant advance, offering new avenues for exploring disease progression and evaluating interventions. In this study, we utilized a mouse model with a Cre-recombinase–inducible *Acta2^R179H^* allele that when activated in smooth muscle cells in heterozygosity recapitulates key aspects of human MSMDS, including vascular steno-occlusive disease, aortic dilation, white matter injury, and bowel and bladder dysfunction (24). This model provides an opportunity to investigate structural and functional alterations in the GI tract resulting from *ACTA2* mutations and lays the groundwork for translational research that could lead to more effective therapeutic strategies for MSMDS.

## Results

### Intestinal dysmotility is a prominent feature in patients with MSMDS.

A retrospective chart review was performed on all patients with MSMDS with R179 variants participating in the Young Genetic Stroke Alliance (YGSA) natural history and biomarkers studies at our institution. This review comprised 24 patients, representing the largest cohort reported to date (Supplemental Table 1; supplemental material available online with this article; https://doi.org/10.1172/jci.insight.190469DS1). Of all patients, 63% were female and ages ranged from 6 months to 26 years. The R179H mutation was present in 88% of patients, and the remainder of patients had R179C. Constipation severe enough to require bowel management, consisting of oral laxatives with or without rectal enemas, was present in 75% of patients. Six patients (25%) were on a prokinetic agent for delayed gastric emptying, intestinal dysmotility, or chronic constipation. Enteral nutrition was required by 50% of patients, defined as partial or complete reliance on tube feeding.

Symptom burden was further characterized in 14 patients based on caregiver survey data (Supplemental Table 2). Many patients remained symptomatic despite therapy, with 46% reporting a history of constipation lasting 5 years or longer, 54% reporting abdominal pain at least “sometimes,” and 46% reporting feelings of incomplete evacuation. More than half had more than 1 bowel movement per day, but stool consistency was most often loose (Bristol Stool Chart type 6), reflecting the impact of combined medical therapy. Symptom onset often occurred at birth, with congenital mydriasis and patent ductus arteriosus as early manifestations. In older patients, GI symptoms frequently preceded genetic diagnosis by many years. These findings highlight the high incidence and symptomatic burden of GI dysmotility in this patient population.

### ACTA2 mutant mice exhibit reduced lifespan and smooth muscle–specific recombination.

To characterize the overall phenotype of the *Myh11-Cre Acta2^R179Hfl/+^* mouse model (referred to as ACTA2 mice hereafter), we compared them with *Acta2^R179Hfl/+^* littermates (control mice). We first assessed gross appearance and survival. Mutant mice were smaller than littermate controls, with reduced body condition evident by 4–5 weeks of age. Kaplan-Meier survival analysis revealed a marked reduction in lifespan, with death as early as 5 weeks of age. Median survival was 6–7 weeks, and no mutant mice survived beyond 15 weeks. In contrast, all control mice survived to the end of the observation period. Early mortality due to the ACTA2 R179H mutation is consistent with severe, systemic smooth muscle dysfunction (Supplemental Figure 1A).

To confirm that Myh11-Cre–driven recombination and mutant ACTA2 expression were restricted to smooth muscle cells, we performed co-immunolabeling of EGFP with lineage-specific markers in the distal colon. EGFP signal showed complete overlap with α-SMA and Myh11, confirming smooth muscle identity (Supplemental Figure 1B). No colocalization of EGFP was observed with markers for ICCs (C-Kit) (Supplemental Figure 1B) or with enteric neurons (Hu) and glial cells (S-100B) (Supplemental Figure 1B). These findings confirmed that Cre-mediated recombination occurs specifically in smooth muscle cells within the adult colonic muscularis propria, with no off-target reporter activity detected in neuronal, glial, or ICC populations.

To further exclude the possibility that apparent overlap could arise from image alignment artifacts or close spatial apposition between smooth muscle fibers and enteric ganglia, we performed whole-mount longitudinal muscle-myenteric plexus (LMMP) preparations from adult Myh11-Cre Acta2^R179H^ ^fl/+^ mice. LMMP tissues were immunolabeled for Hu, S-100B, and EGFP, and high-resolution confocal *Z*-stack imaging was acquired across the full depth of the myenteric plexus. Three-dimensional reconstructions and orthogonal views demonstrated that Myh11-GFP signal was confined to smooth muscle fibers surrounding myenteric ganglia and did not colocalize with Hu^+^ neuronal cell bodies, nor with S-100B^+^ glial cells, at any *Z*-plane (Supplemental Video 1). These data provide unambiguous 3D confirmation that Myh11-Cre–driven recombination in the adult colon is restricted to smooth muscle cells.

### Gut structural changes in ACTA2 mutant mice.

**Figure 1:**
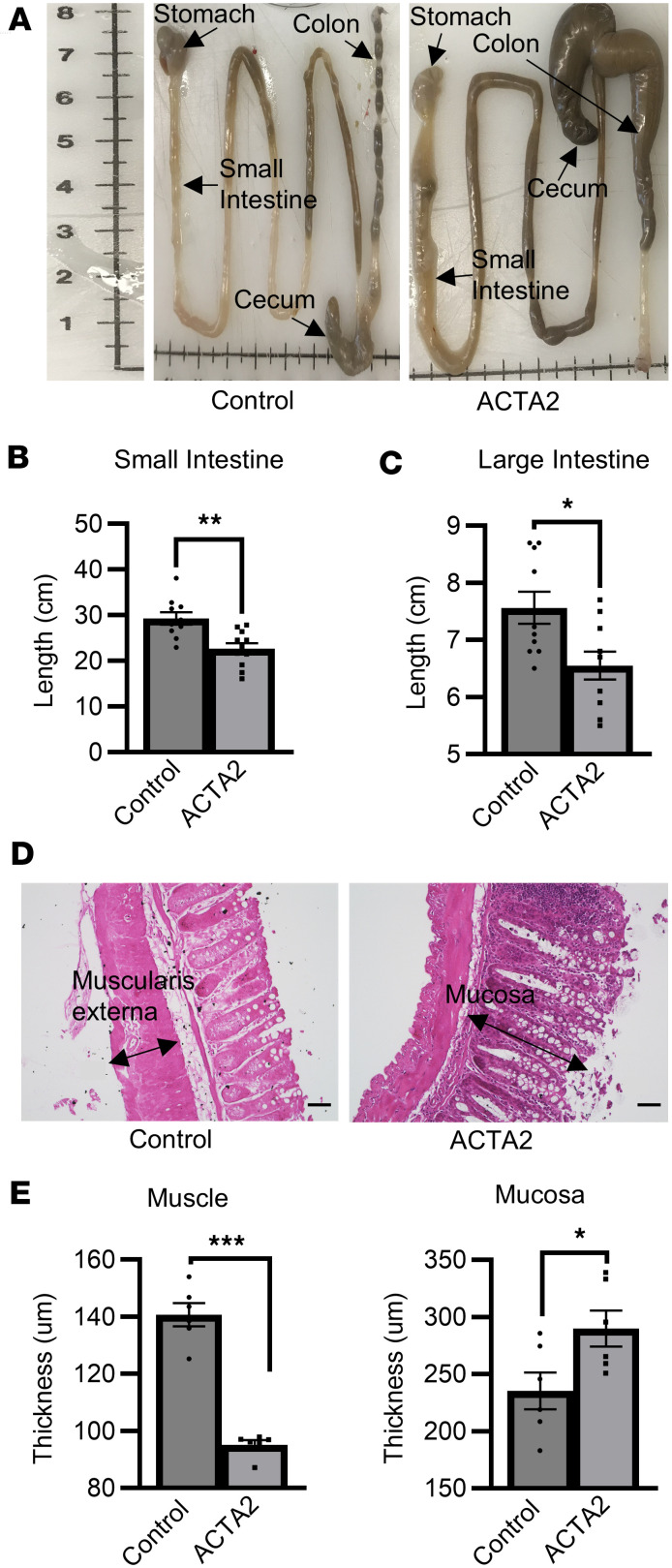
Altered gut architecture in ACTA2 mutant mice. (**A**) Gross morphology of small and large intestine from control and ACTA2 mice, with mutants displaying fecal obstruction/impaction in the cecum and proximal colon. (**B** and **C**) Reduction in the length of the small and large intestine in ACTA2 mice compared with control mice. (**D**) H&E staining of distal colon sections from control and ACTA2 mice. Scale bars: 50 μm. (**E**) Assessment of gut histology revealed thinner muscle layers and thickened mucosa in the distal colon of ACTA2 mice compared with control mice. Each dot represents the average thickness value from 3 measurements (top, middle, and bottom of a representative section) in a single animal. Data are presented as mean ± SEM. Statistical comparisons were made using unpaired 2-tailed *t* tests, as detailed in Methods. *P < 0.05, **P < 0.01, ***P < 0.001.

ACTA2 mice at 6–8 weeks of age exhibited gross distension of the cecum and proximal colon, with a constricted distal colon (Figure 1A). The length of both the small intestine (23.5 ± 1.2 cm) and large intestine (6.6 ± 0.2 cm) was significantly less than control mice (small intestine: 29.3 ± 1.4 cm, *P* < 0.01; large intestine: 7.6 ± 0.3 cm, *P* < 0.05) (Figure 1, B and C). H&E staining of the distalmost 2 cm of colon, which was visibly narrowed in ACTA2 mutant mice, revealed a thinner muscle layer (95.1 ± 1.6 μm) and thicker mucosal layer (289.9 ± 15.7 μm) in ACTA2 mice compared with controls (muscle: 140.7 ± 4.0 μm, *P* < 0.001; mucosa: 235.4 ± 16.2 μm, *P* < 0.05) (Figure 1, D and E). Occasional epithelial disorganization and increased goblet cell content were observed in ACTA2 mutants, suggesting mucosal remodeling secondary to smooth muscle dysfunction.

To further define these regional alterations, we measured the luminal diameters of the proximal, mid, and distal colon following fixation of maximally stretched and pinned colons. ACTA2 mutants exhibited a significant increase in proximal colonic diameter (13.63 ± 0.35 mm) compared with controls (8.67 ± 0.51 mm, *P* < 0.01), and a significant reduction in distal colonic diameter (7.15 ± 0.11 mm) relative to controls (8.93 ± 0.56 mm, *P* < 0.05). Mid-colon diameters were comparable between groups (*P* > 0.05) (Supplemental Figure 1C). These region-specific changes further support segmental remodeling of the colon in ACTA2 R179H mutant mice.

To assess the status of smooth muscle integrity, we performed α-SMA (ACTA2) immunohistochemistry. ACTA2 mutant mice exhibited visibly reduced α-SMA staining intensity and discontinuity in the circular muscle layer of the distal colon compared with controls (Supplemental Figure 1D), consistent with compromised smooth muscle structure and/or reduced α-SMA expression.

### Colonic motility is disrupted in ACTA2 mutant mice.

**Figure 2:**
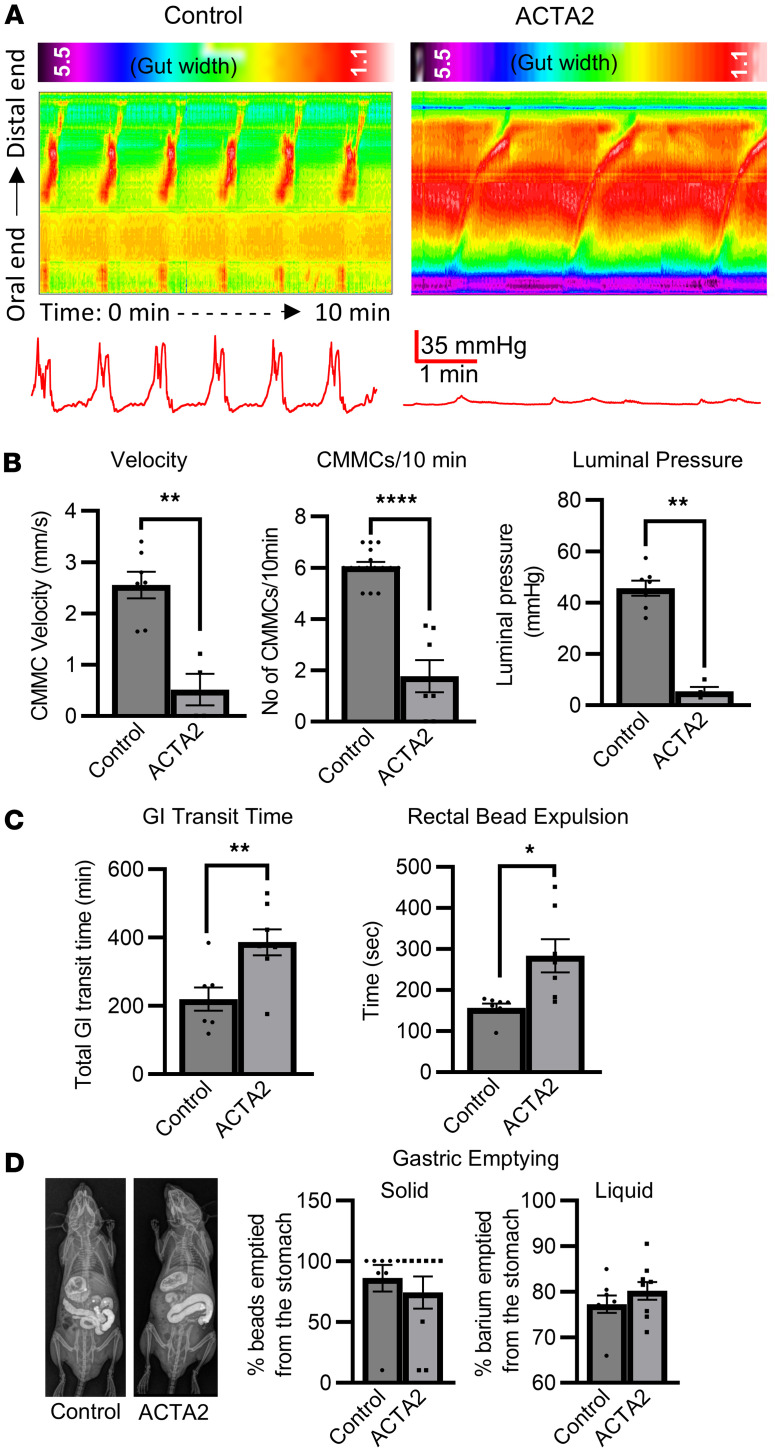
Colonic dysmotility in ACTA2 mutant mice. (**A**) Representative spatiotemporal map kymographs generated from video recordings of colonic motility in control and ACTA2 mice, depicting changes in gut width along the entire length of the colon, with the labeled color scale bar (in mm) representing diameter (cool colors indicating smaller diameter/contraction, warm colors indicating larger diameter/relaxation). Intraluminal pressure represents the maximum pressure (mmHg) recorded during a 10-minute observation period. (**B**) CMMC velocity, frequency, and luminal pressure were significantly decreased in ACTA2 mice compared with control mice. (**C**) Prolonged rectal bead expulsion time in ACTA2 mice. (**D**) Radiographic gastric emptying assay using gavaged radio-opaque beads and contrast showed that both solid and liquid gastric emptying were unaltered in ACTA2 mice. Data are presented as mean ± SEM. Statistical comparisons were made using unpaired 2-tailed *t* tests, as detailed in Methods. *P < 0.05, **P < 0.01, ****P < 0.0001.

To investigate gut motility, kymographs were generated from spatiotemporal maps of colonic motility in 6- to 8-week-old ACTA2 mutant mice and controls. Normal, regular, propagating colonic migrating motor complexes (CMMCs) were observed in control mice (Figure 2A) but were notably absent in ACTA2 mice (Figure 2A). CMMCs were defined as coordinated contractions that propagated in the oral-to-anal direction and extended along at least 50% of the colon length. Although ACTA2 mutants exhibited some irregular contractions, these events lacked sustained propagation and failed to meet the 50% threshold and therefore were not counted as CMMCs. Each CMMC in control mice was associated with an increase in intraluminal pressure (46 ± 3.0 mmHg), whereas ACTA2 mice had markedly attenuated pressure changes (6 ± 1.6 mmHg, *P* < 0.01) (Figure 2A). CMMC velocity and frequency were significantly reduced in ACTA2 mice (CMMC velocity: 0.52 ± 0.3 mm/s, *P* < 0.01; frequency: 1.8 ± 0.6 per 10 minutes, *P* < 0.001) compared with littermate controls (CMMC velocity: 2.6 ± 0.3 mm/s; frequency: 6.1 ± 0.2 per 10 minutes) (Figure 2B).

Further in vivo GI functional analysis demonstrated that gut transit was delayed in ACTA2 mice, as evidenced by prolonged whole-gut transit time (386.1 ± 38.1 minutes, *P* < 0.01), measured by the expulsion time of carmine red–containing fecal pellets compared with controls (219.7 ± 34.2 minutes) (Figure 2C). Additionally, rectal bead expulsion time was also significantly prolonged in ACTA2 mice (283.8 ± 40.6 minutes, *P* < 0.05) compared with the control (156.5 ± 10.8 minutes) (Figure 2C). Radiographic assessment of gastric emptying using gavaged radio-opaque beads and contrast revealed that both solid and liquid gastric emptying were unaltered in ACTA2 mice (Figure 2D).

### Smooth muscle contractile responses are attenuated in ACTA2 mutant mice.

To investigate the mechanisms underlying gut dysmotility in ACTA2 mutant mice, smooth muscle contractility in the distal colon was assessed using electrical field stimulation (EFS), acetylcholine (ACh), and the global depolarizing agent potassium chloride (KCl) using standard organ bath techniques. Colons from ACTA2 mutants displayed striking spontaneous activity, marked by rhythmic, high-amplitude contractions that persisted throughout the recordings. Such activity was absent in controls (Supplemental Figure 2A). Quantitative analysis confirmed this abnormal phenotype, with ACTA2 mutants showing significantly greater baseline contractile activity (AUC 176.5 ± 50.5 g × s) compared with controls (AUC 36.9 ± 11.2 g × s, *P* < 0.001, Supplemental Figure 2B), indicating both enhanced magnitude and sustained rhythmicity of spontaneous contractions.

**Figure 3:**
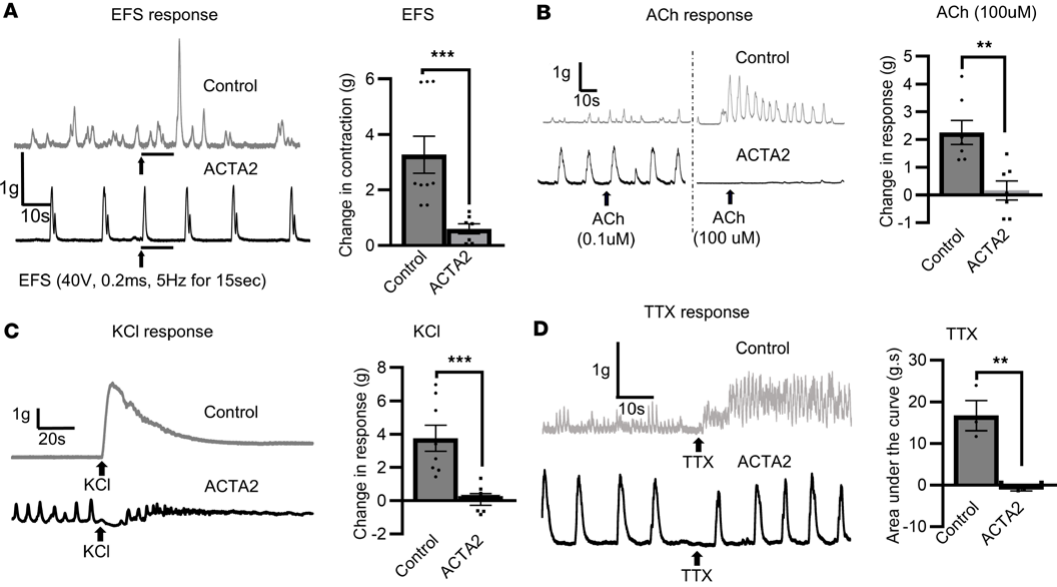
Reduced smooth muscle contraction in ACTA2 mutant mice in response to EFS, ACh, and KCl (distal colon). (**A**–**C**) Representative traces of smooth muscle contraction in response to EFS (**A**), ACh (**B**), and KCl (**C**) in distal colon rings from control and ACTA2 mice. Bar graphs depict the maximum contractile force (g) recorded within 5 minutes after stimulus addition, compared with the maximum value during the 60-second baseline period prior to stimulation. This difference was used to calculate the absolute change in response (Δg). (**A**) Application of EFS caused significant muscle contraction in the distal colon of control mice but no response in ACTA2 mice. Maximum effects are shown as the absolute change from basal values. (**B**) Both low and high doses of ACh failed to induce contraction in ACTA2 mice (left panel, representative trace). (**C**) Muscle contraction in response to KCl was also attenuated in ACTA2 mice. (**D**) TTX increased the frequency and amplitude of tonic contractions in control mice but had no effect on tissues from ACTA2 mice. Data are presented as mean ± SEM. Statistical comparisons were made using unpaired 2-tailed *t* tests, as detailed in Methods. **P < 0.01, ***P < 0.001.

When EFS was applied, robust muscle contractions were observed in control colon (3.3 ± 0.7 g), but these contractions were significantly reduced in amplitude in ACTA2 mice (0.6 ± 0.2 g, *P* < 0.001, Figure 3A).

To assess cholinergic receptor–mediated contractility, we generated a full dose–response curve to Ach (0.01–100 μM) in colonic smooth muscle from control and ACTA2 mutant mice (Supplemental Figure 2C). Control tissues exhibited the expected graded, dose-dependent increase in contractile force, culminating in robust contractions at the highest concentration (~2.3 g at 100 μM). In contrast, ACTA2 mutant tissues showed a profoundly blunted and heterogeneous profile. Some mutants displayed only small, incremental increases in force (Supplemental Figure 2, trace i), while most showed little to no response (trace ii), and a subset even exhibited paradoxical reductions in tone at higher doses (trace iii). This paradoxical effect likely reflects suppression of the spontaneous rhythmic contractions that are prominent in ACTA2 colon. As a result, the group dose-response curve in ACTA2 mutants remained essentially flat, in stark contrast to the steep upward trajectory observed in controls (Supplemental Figure 2D). Figure 3B provides representative traces at the lowest (0.1 μM) and highest (100 μM) ACh concentrations. In control colon, 0.1 μM ACh produced no effect, whereas 100 μM triggered strong phasic contractions. In ACTA2 mutants, high-dose stimulation was frequently accompanied by suppression of baseline rhythmic activity rather than contraction. Quantification confirmed a significant reduction in peak contractile force in ACTA2 mutants (0.2 ± 0.3 g) compared with controls (2.3 ± 0.4 g, *P* < 0.01, Figure 3B).

To assess whether the high-dose ACh response reflected a tonic contraction or impaired relaxation, we performed a complementary dose–response analysis using sodium nitroprusside. In both control and ACTA2 mutant tissues, sodium nitroprusside induced dose-dependent relaxation, with a noticeable decrease in basal tone beginning at low concentrations and near-complete abolition of spontaneous activity at doses of 50 μM or higher (Supplemental Figure 2, E and F). These findings indicate that the smooth muscle in ACTA2 mutants retains the capacity to relax in response to nitric oxide signaling and that the suppression of phasic activity seen with high-dose ACh likely reflects a failure in excitatory contractile signaling, rather than abnormal hypercontractility or sustained tetany.

To assess receptor-independent smooth muscle excitability, we tested responses to potassium chloride (KCl). In control tissue, KCl induced strong contractions (5.8 ± 0.6 g), while ACTA2 mutant muscle showed significantly reduced responses (0.8 ± 0.4 g, *P* < 0.001) and loss of organized phasic activity (Figure 3C).

We also investigated whether these impairments were region specific by assessing EFS-induced contractions in the mid and proximal colon and showed that in ACTA2 mutants, contractile responses were significantly reduced in the mid-colon (0.2 ± 0.3 g; *P* < 0.01 vs. control) but were not significantly different from controls in the proximal colon (0.6 ± 0.1 g; *P* = nonsignificant) (Supplemental Figure 2G). These findings reveal a region-dependent gradient of smooth muscle dysfunction in ACTA2 mutant mice, with impairments most severe in the distal colon and progressively less pronounced in more proximal segments. Consistent with this observation, ileal muscle contractile responses to EFS, ACh, and KCl were not significantly different between ACTA2 mutants and controls (data not shown).

As noted above, a consistent feature of gut contractility in ACTA2 mice is the occurrence of spontaneous, regular contractions (Figure 3, A–C). To test whether this contractile activity is mediated by enteric neurons, tetrodotoxin (TTX) was added to the organ bath. In the presence of TTX, the tonic contractile activity in control mice became more frequent and of higher amplitude. However, there was no change in the spontaneous muscle contractions in ACTA2 mice, as shown in Figure 3D.

### Assessment of ENS and other cell types in ACTA2 mutant mice.

**Figure 4:**
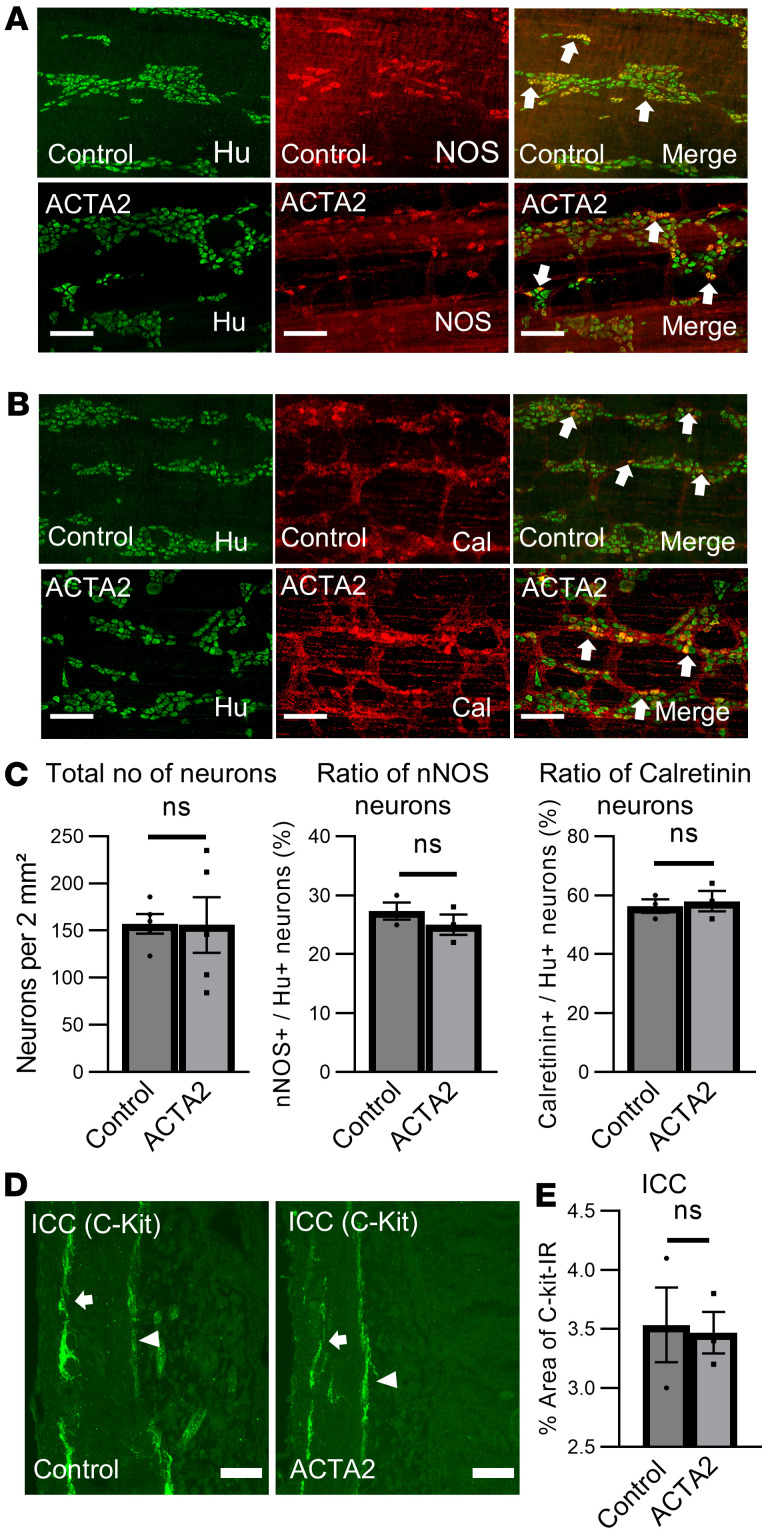
Enteric nervous system in ACTA2 mutant mice. (**A** and **B**) Morphometric analysis of the colonic myenteric plexus stained with neuronal markers Hu (total neurons), nNOS (inhibitory motor neurons), and calretinin (commonly associated with sensory neurons, interneurons, and some excitatory motor neurons). (**C**) Quantification of Hu^+^, nNOS^+^, and calretinin^+^ neurons per 2 mm^2^ area in whole-mount myenteric plexus preparations. Bar graphs on the right show the percentage of Hu^+^ neurons that were also positive for nNOS or calretinin. (**D**) Confocal images of ICC in the distal colon labeled with C-Kit. ICCs located at the myenteric plexus (ICC-MY) are indicated with arrows, and those near the submucosa (ICC-SM) with arrowheads. (**E**) Quantification of total C-Kit^+^ ICC immunoreactivity in control and ACTA2 mice. Scale bars: 50 μm. Data are presented as mean ± SEM. Statistical comparisons were made using unpaired 2-tailed *t* tests, as detailed in Methods.

We performed immunohistochemical analysis to evaluate potential changes in the ENS of ACTA2 mutant mice. No significant differences were observed in the total number of Hu^+^ myenteric neurons or in the proportion of Hu^+^ neurons expressing nNOS (nitrergic) or calretinin (cholinergic) compared with control mice (Figure 4).

Together, these data demonstrate that ACTA2 mutations do not cause overt loss or structural disruption of enteric neurons. However, single-nuclei RNA-seq (snRNA-seq) analysis revealed modest changes in the expression of several neuronal and glial genes (e.g., *Tac1*, *Vip*), suggesting that although the structural framework of the ENS was preserved, transcriptional adaptations within these cell populations may accompany the broader smooth muscle dysfunction.

### Gene expression is altered in the colonic muscularis propria of ACTA2 mutant mice.

**Figure 5:**
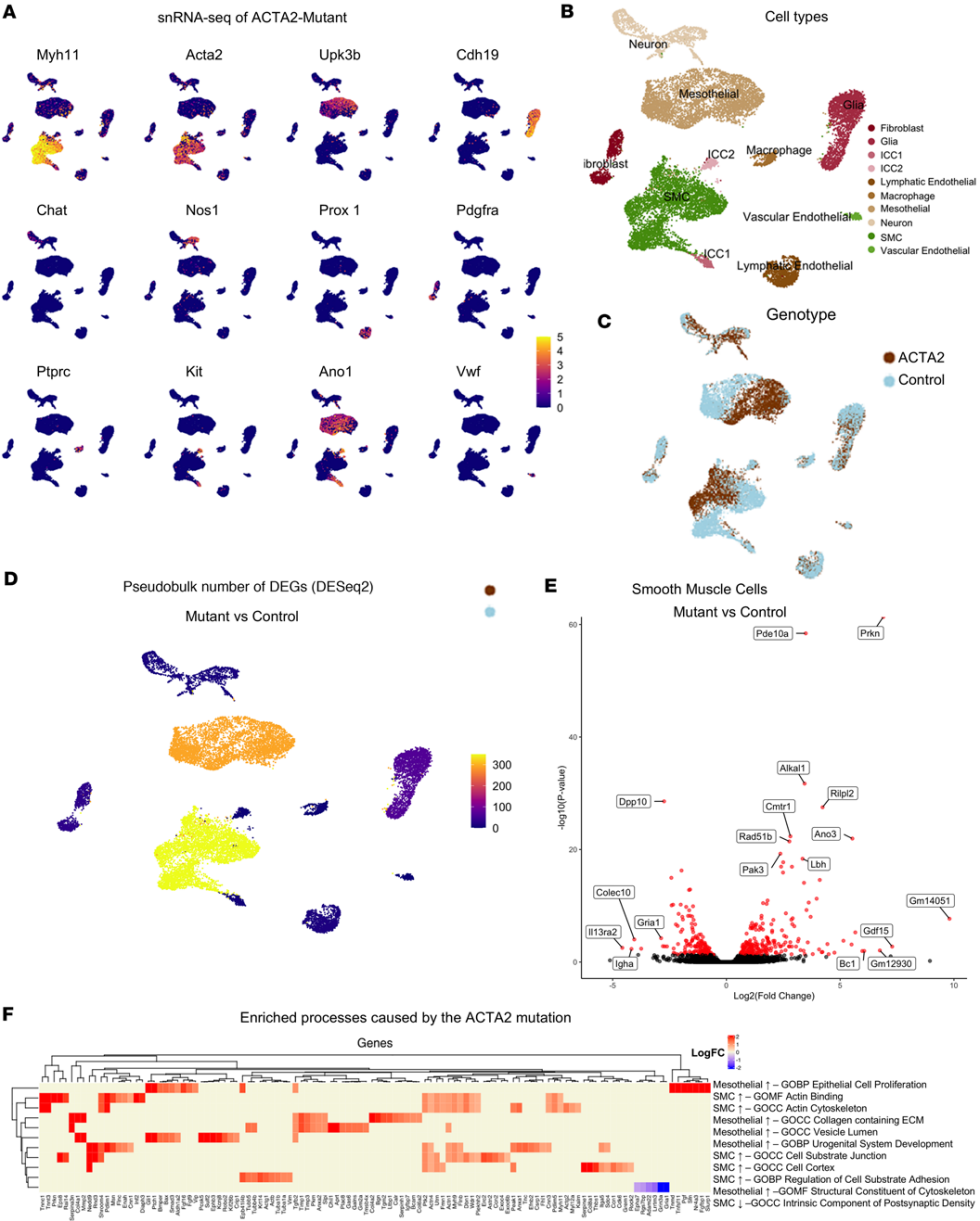
Transcriptional changes in intestinal smooth muscle cells in ACTA2 mutant mice. (**A**) Expression of gene markers for smooth muscle cells (*Myh11* and *Acta2*), mesothelial cells (*Upk3b*), enteric glial cells (*Cdh19*), excitatory and inhibitory enteric neurons (*Chat* and *Nos1*), lymphatic endothelial cells (*Prox1*), fibroblasts (*Pdgfra*), immune cells (*Ptprc*), ICCs (*Kit* and *Ano1*), and vascular endothelial cells (*Vwf*) in nuclei isolated from the muscularis propria of the colon from control and ACTA2 mutant mice represented as UMAP plots. (**B** and **C**) Unsupervised clustering of cell populations annotated for cell type identity (**B**) and genotype (**C**) in UMAP space. (**D**) Total number of differentially expressed genes (DEGs) between ACTA2 mutant mice and controls for each cell population cluster. (**E**) Volcano plot showing DEGs in smooth muscle cells from ACTA2 mutants compared with controls. The *x* axis indicates log_2_ fold change (mutant vs. control), with positive values (right) representing genes upregulated in ACTA2 mutants and negative values (left) representing genes downregulated in mutants. The *y* axis indicates the –log_10_ adjusted *P* value. Red dots denote significantly differentially expressed genes (adjusted *P* < 0.05), and black dots represent nonsignificant genes. Selected genes of interest are labeled. (**F**) Overrepresentation analysis of DEGs between ACTA2 mutant mice and controls against the GO database. Heatmap shows the logFC changes in mutants compared with controls for each gene associated with the gene ontology.

We performed snRNA-seq on control and ACTA2 mutant mice, focusing specifically on the muscularis propria of the colon. The analysis captured smooth muscle cells (*Myh11*, *Acta2*, *Actg2*), mesothelial cells (*Upk3b*), enteric glial cells (*Cdh19*, *Plp1*), enteric neurons (*Elavl4*, *Chat*, and *Nos1*), lymphatic endothelial cells (*Prox1*, *Reln*), fibroblasts (*Lama2*, *Pdgfra*, *Svep1*), macrophages (*Ptprc*, *Mrc1*), ICCs (*Kit* and *Ano1*), and vascular endothelial cells (*Vwf*, *Pecam1*) (Figure 5A). Full differential expression results for all annotated clusters are provided in Supplemental Figure 3 and Supplemental Table 3.

All cell populations were present in both control and mutant colons (Figure 5, B and C). In addition to genotype-based segregation (Figure 5C), we examined whether cell clustering patterns were influenced by sex. Uniform manifold approximation and projection (UMAP) visualization colored by sex revealed no clear separation of clusters between male and female mice, with both sexes represented across all cell populations (Supplemental Figure 4). This aligns with our statistical analyses showing no significant sex-based differences in the measured anatomical or molecular parameters. Differential gene expression analysis was conducted across all identified cell populations. Smooth muscle cells exhibited the greatest number of differentially expressed genes (DEGs; 213 upregulated and 136 downregulated) in mutant compared with control mice, consistent with the known role of ACTA2 in smooth muscle cell function (Figure 5, D and E, and Supplemental Tables 4 and 5). Interestingly, we also observed gene expression changes in mesothelial cells (161 upregulated and 130 downregulated in mutants) (Figure 5D). Given that mesothelial cells do not express *Acta2*, this suggests that non–smooth muscle cell types may exhibit important alterations secondary to the effects of smooth muscle dysfunction. Overrepresentation analysis was performed for each cell type separately. Genes upregulated and downregulated in ACTA2 mutants versus controls were analyzed independently. Enrichment for Gene Ontology (GO) terms was only observed in smooth muscle cells and mesothelial cells (Figure 5F). This analysis confirmed that upregulated genes in ACTA2 mutant smooth muscle cells were enriched for pathways involving actin binding, cytoskeletal organization, and cell-substrate adhesion (Supplemental Table 6). Among the most significantly upregulated genes in smooth muscle cells of ACTA2 mutant mice were *Pde10a* and *Prkn* (Figure 5E). *Pde10a* showed a greater than 3-fold increase in expression (avg log_2_FC = 3.32, adjusted *P* = 1.58 × 10^–184^), with expression detected in 45.1% of mutant smooth muscle nuclei compared with 9.9% in controls. *Prkn*, a gene involved in mitochondrial homeostasis and cellular stress response, was even more enriched (avg log_2_FC = 6.74, adjusted *P* = 0), expressed in 83% of mutant smooth muscle cells versus 30% in controls. This suggests that ACTA2 mutation leads to transcriptional activation of genes associated with cell stress, metabolism, and remodeling pathways.

Although smooth muscle cells had the most pronounced transcriptional changes in ACTA2 mutants (217 upregulated and 141 downregulated genes), other cell types also exhibited measurable alterations (Supplemental Figure 5 and Supplemental Tables 4 and 5). Glial cells displayed 48 upregulated and 18 downregulated genes; fibroblasts had 22 upregulated and 9 downregulated genes; neurons exhibited 12 upregulated genes; lymphatic endothelial cells had 6 upregulated and 9 downregulated genes; and ICC1 and ICC2 clusters exhibited very few DEGs (1 upregulated and 4 downregulated and 1 upregulated/1 downregulated, respectively). These findings indicate that ACTA2 mutation leads to broader transcriptional effects across multiple cell types within the colonic muscularis propria, though the magnitude of change is greatest in smooth muscle cells.

### Cross-tissue integration of intestinal and vascular smooth muscle datasets reveals conserved and tissue-specific effects of ACTA2 R179 mutations.

To compare the transcriptional effects of ACTA2 mutations across tissues, we integrated our intestinal snRNA-seq data with the aortic vascular smooth muscle cell dataset from Kwartler et al., 2023 (25). The combined UMAP showed that intestinal and vascular smooth muscle cells share a common transcriptional space while retaining tissue-specific subclusters (Supplemental Figure 6A). Smooth muscle identity was confirmed by expression of Myh11, Acta2, and Actg2, with no contamination from other lineages (Supplemental Figure 6B).

Contractile module scores were reduced in ACTA2 mutant vascular smooth muscle cells but preserved in intestinal smooth muscle cells, while proliferative module scores were elevated only in vascular smooth muscle cells (Supplemental Figure 7, A and B). Among shared DEGs, 76.3% showed the same direction of regulation (Supplemental Figure 7C). Genes upregulated in ACTA2 mutants were enriched for actin cytoskeleton and adhesion pathways, whereas genes upregulated in controls were linked to extracellular matrix organization (Supplemental Figure 7D). These findings highlight both conserved and tissue-specific transcriptional effects of ACTA2 mutations, with distinct remodeling mechanisms in intestinal versus vascular smooth muscle.

### Validation of snRNA-seq findings for PDE10a and Prkn.

To validate the differential expression of *Pde10a* and *Prkn* identified in our snRNA-seq analysis, we examined protein-level changes using quantitative immunofluorescence in the distal colonic muscularis propria of ACTA2 mutant and control mice. These genes were selected because they represent 2 of the most significantly altered transcripts in intestinal smooth muscle clusters and are implicated in smooth muscle signaling (*Pde10a*) and mitochondrial homeostasis (*Prkn*).

Consistent with the transcriptomic findings, PDE10A MFI was significantly elevated in ACTA2 mutants compared with controls (1.5 ± 0.1 vs. 0.9 ± 0.1, *P* < 0.05). Parkin immunoreactivity was also significantly increased in mutants (1.8 ± 0.1 vs. 1.2 ± 0.1, *P* < 0.05) (Supplemental Figure 8), with staining largely localized to smooth muscle, although the extent of cell type specificity may vary. These findings support increased PDE10a and Parkin expression in ACTA2 mutant colonic smooth muscle, consistent with the snRNA-seq data, while acknowledging contributions from other cell types in the case of PDE10a.

### Colonic immune cell populations are perturbed in ACTA2 mutants.

To determine whether the functional changes in ACTA2 colon are associated with alterations in immune cell populations, immunohistochemistry was performed on full-thickness colon sections. Staining for the pan-leukocyte marker CD45 (Supplemental Figure 9A) revealed higher numbers of immune cells in the colon of ACTA2 mutant mice compared with littermate controls (Supplemental Figure 9B), consistent with enhanced immune cell infiltration. To quantify relative changes in lymphoid and myeloid populations, immunophenotyping was performed on full-thickness colon tissue from ACTA2 mice and littermate controls by flow cytometry. ACTA2 mice had higher proportions of CD45^+^ immune cells within the colon (mean 30.7% vs. 9.67%, *P* = 0.003), consistent with colitis (Supplemental Figure 9, C and D). Further characterization of lymphoid and myeloid subpopulations was performed using the gating strategy (outlined in Supplemental Figure 9E) to isolate live, single CD45^+^ immune cells. We observed a trend toward increased colonic CD8^+^ T cells in ACTA2 mice (Supplemental Figure 9, F and G) compared with littermate controls (mean 1.11% vs. 0.96%, *P* = 0.58), with a statistically significant expansion within the CD3^+^ T cell compartment (mean 8.03% vs. 5.60%, *P* = 0.047; Supplemental Figure 9G). The proportion of CD4^+^ T cells, amongst all colonic immune cells and within the T lymphocyte compartment, was preserved between groups (Supplemental Figure 9H). We next characterized the myeloid compartment of the colon, identifying macrophages as CD11b^+^F4/80^+^ cells (Supplemental Figure 9I) and neutrophils as CD11b^+^Ly6c^hi^Ly6G^+^ cells. We observed down-trending numbers of colonic macrophages (Supplemental Figure 9J) throughout our studies, a major resident population of the colon under physiological conditions. There was no significant neutrophilic infiltration in the colon of ACTA2 mice (mean 1.19% vs. 1.25%, *P* = 0.945; Supplemental Figure 9K). Overall, our findings were consistent with elevated lymphocytic infiltration in the colon of ACTA2 mice.

## Discussion

MSMDS, a rare smooth muscle disorder caused primarily by missense pathogenic variants at arginine 179 of the ACTA2 gene, is associated with thoracic aortic aneurysms, cerebrovascular abnormalities, congenital mydriasis, hypotonic bladder, and intestinal dysmotility. Upon review of our single-institution cohort of 24 patients with MSMDS, the largest such group reported with respect to GI symptoms, we found a high prevalence of significant, chronic constipation requiring medications, in addition to a high rate of patients requiring enteral nutrition and prokinetic drugs. These GI manifestations are significant contributors to morbidity and reduced quality of life, underscoring the need for mechanistic insights and novel therapeutic strategies (4–10), and providing the impetus for this study.

### Structural and functional deficits in ACTA2 mutants.

We used the ACTA2 R179H mouse model to assess the structural and functional defects associated with MSMDS in the GI tract. We identified major structural abnormalities, including narrowing of the distal colon with proximal dilatation. This was associated with changes in muscle wall thickness, indicative of smooth muscle remodeling. Immunohistochemistry showed visibly reduced α-SMA staining in the distal colon of ACTA2 mutants, but we did not directly assess whether this thinning resulted from reduced smooth muscle cell number, smaller cell size, or both. Future studies incorporating cell-specific quantification and morphometric analysis will be needed to resolve this question. In addition to muscle remodeling, we observed epithelial irregularities and goblet cell expansion in the distal colon of ACTA2 mutants, suggesting that smooth muscle dysfunction may indirectly influence mucosal architecture. Although ACTA2 mutant mice showed markedly reduced intraluminal pressure during CMMCs, the distal colon remained narrowed. This paradox may reflect tonic contraction or maladaptive structural remodeling of the distal smooth muscle, rather than active, high-pressure contractions. Segmental proximal dilation likely results from accumulation of luminal contents due to failed propulsion, further supporting the notion of region-specific dysmotility. An alternative explanation for the distal narrowing is that reduced propulsive force in the mid-colon limits delivery of stool to the distal segment. Underfilling may restrict distension-driven growth and compliance, resulting in a relatively narrow distal lumen, similar to the microcolon phenotype described in visceral myopathy. This mechanism may act in parallel with intrinsic smooth muscle tone and cytoskeletal changes to shape the regional caliber pattern in ACTA2 mutant colon.

Notably, isolated colonic rings from ACTA2 mice displayed prominent spontaneous myogenic contractions despite this low intraluminal pressure in intact tissue. This suggests that individual muscle segments can still generate force, but the contractions are poorly coordinated and fail to translate into effective luminal pressurization or propulsion. In other words, the muscle can contract, but it cannot contract together. The lack of response to TTX further supports impaired ENS-smooth muscle communication, with preserved intrinsic rhythmicity but loss of coordinated motor pattern generation. These findings are consistent with studies highlighting the central role of actin cytoskeletal disruption in the development of visceral myopathy (8, 21). The GI morphological changes we observed were accompanied by functional deficits in colonic motility, characterized by prolonged intestinal transit time, absence of CMMCs, and delayed rectal bead expulsion times.

We also assessed whether sex influenced the ACTA2 phenotypes. Despite the female predominance in the clinical cohort, male and female mice showed no significant differences in intestinal measurements, motility assays, or smooth muscle contractility (*P* > 0.05). snRNA-seq similarly showed no sex-based clustering. These findings suggest comparable effects in both sexes, though subtle differences cannot be excluded.

Single-cell transcriptomics and organ bath studies confirmed smooth muscle dysfunction in the ACTA2 mutant mice, with markedly reduced colonic contractile responses to electrical (EFS) and pharmacological (ACh, KCl) stimulation. Interestingly, contractile responses in the ileum were not significantly different between ACTA2 mutants and controls, suggesting region-specific effects of the mutation on visceral smooth muscle function. As noted above, despite the reduction in GI colonic contractile response, ACTA2 mice exhibited spontaneous rhythmic colonic contractions. This pattern suggests that the R179H mutation may preferentially impair pathways involved in evoked contractility, while intrinsic myogenic rhythmicity is preserved. As our model expresses both WT and mutant α-SMA, these findings likely reflect a functional disruption of excitation-contraction coupling rather than the absolute requirement of α-SMA for provoked responses. Further studies are needed to determine whether distinct actin cytoskeletal networks or signaling pathways underlie the differential effects on spontaneous versus evoked contractions. Smooth muscle contraction relies on both electromechanical and pharmaco-mechanical coupling, mediated by L-type voltage-gated calcium channels and signaling pathways such as Rho kinase and myosin light chain kinase (26–28). In the context of the R179H mutation, destabilization of the cytoskeletal framework may impair the transduction of intracellular calcium flux into mechanical force, as suggested by the blunted response to KCl-induced depolarization. This supports a defect in excitation-contraction coupling mechanisms downstream of depolarization, rather than a complete absence of α-SMA–mediated contractile capacity. Moreover, distinct pathophysiological mechanisms appear to underlie the reduced responses to neurogenic and pharmacological stimuli, with neuronal signaling deficits impairing communication and cytoskeletal disruptions affecting direct smooth muscle contractility (29). Further studies will be essential to directly test these possibilities, including detailed analyses of actin polymerization dynamics, intracellular calcium flux, and voltage-dependent calcium channel function in ACTA2 mutant smooth muscle cells.

The differential effects of TTX on colonic contractility we observed further highlight the decoupling of enteric neuronal inputs from smooth muscle cells in ACTA2 mutants. In control mice, TTX increased the frequency and amplitude of contractions, suggesting that tonic contractile activity is under inhibitory neuronal control, likely mediated by nitrergic pathways. This is consistent with previous findings showing that TTX enhances colonic contractility by inhibiting nitrergic neural input, which normally suppresses smooth muscle cell activity (30, 31). The lack of a similar response in ACTA2 mutants indicates that enteric inhibitory neurons fail to communicate with smooth muscle cells, consistent with their inability to mediate contractile responses to ACh, EFS, or KCl. This supports the notion that spontaneous activity in the ACTA2 mutant colon is entirely myogenic in origin, driven by intrinsic smooth muscle mechanisms and the pacemaker activity of ICCs within the SIP syncytium, rather than by neurogenic modulation.

Another important factor to consider for elucidating the changes in colonic contractions in ACTA2 mice is γ-actin (in humans encoded by *ACTG2*), the predominant actin isoform in visceral smooth muscle. Although ACTA2 mutations disrupt α-SMA, ACTG2 may compensate by preserving intracellular actin dynamics and stabilizing the cytoskeletal framework. In addition, all cells contain 2 ubiquitous cytoskeletal actins, β-actin (ACTB) and cytoplasmic γ-actin (ACTG1), which contribute to general cellular architecture and may provide further baseline structural support in smooth muscle cells (32). This redundancy could support residual contractile activity, even in the face of broader cytoskeletal dysfunction (33). Together, ICC activity and potential compensatory mechanisms of γ-actin highlight the multifaceted regulation of GI motility and may explain the seeming paradox in the preservation of rhythmic contractile activity in ACTA2 mutant mice, although further studies are needed to address this issue.

### Gene expression changes.

Our snRNA analysis of the colon in ACTA2 mutant and control mice identified substantial changes in intestinal smooth muscle cells at the transcriptional level. The DEGs in intestinal smooth muscle cells were associated with actin binding and actin cytoskeleton pathways, supporting the hypothesis that disruptions in the actin cytoskeleton drive the smooth muscle dysfunction observed. Notably, transcriptional alterations were also observed in other cell types, with mesothelial cells showing the most pronounced changes. As these cells do not express *Acta2*, these changes may be considered secondary, which could be caused by factors such as intestinal stretch, given the megacolon-like phenotype in these mice. This finding highlights that disruption to the homeostasis of smooth muscle cell signaling and/or function can impact other cell types, changing their properties at a transcriptional level. The implications of these secondary effects in non–smooth muscle cells in MSMDS are unknown but could be involved in the progression of the disease and warrant further consideration.

Among the most upregulated genes in ACTA2 mutant smooth muscle cells was *Pde10a*, a phosphodiesterase known to promote smooth muscle proliferation and remodeling. This gene, which was expressed in nearly half of mutant smooth muscle cells compared with only 10% in controls, has been previously implicated in pathological vascular remodeling by antagonizing cGMP/PKG signaling (34). Its upregulation in ACTA2 R179H colon may reflect an aberrant compensatory mechanism in response to impaired actin cytoskeleton function, potentially contributing to maladaptive remodeling or failed contractile adaptation in the gut. This finding highlights the complexity of downstream molecular responses in smooth muscle cells and suggests that, beyond structural actin defects, secondary signaling pathways such as PDE10A-driven remodeling may amplify the dysfunction. In addition to smooth muscle cells, *Pde10a* upregulation was also detected in mesothelial, glial, and fibroblast clusters, indicating that its induction may represent a broader adaptive response to tissue stress rather than a smooth muscle–restricted driver. Prior studies have shown *PDE10A* expression in enteric neurons of the adult gut (35), and more recent work demonstrated *PDE10A* expression in multiple enteric and non-neuronal cell types, including within myenteric ganglia and epithelial crypts (36). *PDE10A* dysregulation may therefore result from altered signaling responses across multiple cell types in the context of smooth muscle dysfunction.

Our observation that ACTA2 mutant mice exhibit changes in colonic T cell subsets, including expansion of CD8^+^ T cells within the CD3^+^ compartment, likely reflects secondary immune modulation driven by smooth muscle dysfunction rather than a direct effect on lymphocytes. Smooth muscle cells are increasingly recognized as active participants in mucosal immunity; they can influence immune responses through the production of cytokines and chemokines and even through antigen presentation capabilities. For example, smooth muscle cells treated with IFN-γ upregulate MHC expression and can directly promote T cell proliferation (37). Additionally, smooth muscle myocytes express inflammatory mediators such as IL-6, TNF-α, and IL-1β, which are well known to recruit and activate immune cells in the gut mucosa (38, 39). Disruption of the actin cytoskeleton in ACTA2 mutant smooth muscle cells could impair epithelial-mesenchymal crosstalk and extracellular matrix integrity, creating tissue stress signals that enhance T cell recruitment. Recent work also shows that abnormal cyclic stretch induces a synthetic and proinflammatory phenotype in human intestinal smooth muscle cells, including cytokine upregulation and altered signaling to neighboring cells, reinforcing the idea that mechanical stress and smooth muscle dysfunction can actively modulate immune responses (40). Furthermore, altered motility and luminal dynamics may increase mucosal exposure to microbial antigens, promoting expansion of mucosal T cell populations. Overall, these findings suggest that structural and functional defects in smooth muscle cells can indirectly reshape the immune landscape of the colon, providing a plausible mechanistic link between mesenchymal dysfunction and mucosal immune dysregulation.

In summary, the ACTA2 R179H mouse model provides a comprehensive platform for studying the interplay between cytoskeletal dysfunction and GI dysmotility in MSMDS. By delineating the structural and functional mechanisms underlying these defects, this model offers new insights into the pathophysiology of intestinal dysmotility and advances our understanding of MSMDS, paving the way for potentially novel strategies to mitigate smooth muscle dysfunction and improve outcomes in this disorder.

## Methods

### Sex as a biological variable

To assess potential sex differences, we analyzed male and female mice separately for intestinal length, tissue morphology, whole-gut transit time, and smooth muscle contractility. Statistical analyses did not reveal any significant sex-based differences across these parameters (*P* > 0.05). Therefore, data from both sexes were combined in the final analyses to improve statistical power and clarity.

### Patients

After obtaining IRB approval, a retrospective chart review, focusing specifically on presence of symptoms related to GI dysmotility, was performed of all patients with ACTA2 R179 pathogenic variants causing MSMDS participating in the YGSA natural history and biomarkers study housed at Massachusetts General Hospital. A total of 24 patients were identified, and the results are summarized in Supplemental Table 1.

### Animals

All animal procedures were approved by the IACUC of Massachusetts General Hospital (protocols 2018N000067 and 2013N000115). The *Acta2^R179Hfl/+^* line was generated via homology-directed repair to introduce the R179H mutation into embryonic stem cells. Correctly targeted clones were microinjected into C57BL/6 albino blastocysts and transferred to CD-1 pseudo-pregnant females (Cyagen) (24).

To achieve smooth muscle–specific recombination, Acta2^R179Hfl/fl^ mice were bred with Myh11-Cre transgenic mice [B6.Cg-Tg(Myh11-Cre-EGFP)2Mik/J; The Jackson Laboratory, 007742]. Offspring expressing the mutant allele in heterozygosity (*Myh11-Cre Acta2^R179Hfl/+^*, hereafter “ACTA2 mice”) were compared with *Acta2^R179Hfl/+^* littermates (“controls”).

Genotyping was performed using DNA from toe tissue collected at postnatal day 8–10. Adult mice (both sexes, 6–8 weeks old) were used for all experiments.

Mice were bred and maintained under specific pathogen–free husbandry conditions at the Massachusetts General Hospital Center for Comparative Medicine in individually ventilated cages (≤4 mice/cage) with autoclaved corn cob bedding and nesting material. The facility maintained a 12-hour light/12-hour dark cycle (lights on 6:00 am), temperature of 20°C–22°C, and 40%–60% relative humidity. Mice had ad libitum access to autoclaved water and irradiated chow (LabDiet 5053, PicoLab Rodent Diet 20). All cages were changed weekly under specific pathogen–free husbandry conditions.

### Histology

For H&E staining, paraffin-embedded colonic samples were sectioned at 5 μm, deparaffinized, cleared, and rehydrated in graded solutions as previously described (41). Distal colon segments (last ~2 cm) were used for thickness measurements. Muscle and mucosal thickness were measured in ImageJ (NIH) on calibrated 20× images. Three nonoverlapping sites (top, middle, bottom) were averaged per animal, and each dot in the graphs represents 1 animal.

For luminal diameter analysis, the full colon was gently dissected, maximally stretched, and pinned flat on a Sylgard-coated dish to standardize shape, then fixed in 4% paraformaldehyde overnight at 4°C. A calibrated ruler was placed adjacent for imaging, and diameters were measured in ImageJ (NIH) at defined sites: proximal (~2 cm from cecum), mid (midpoint between cecum and anus), and distal (last 2 cm).

### Immunohistochemistry

Immunohistochemistry for characterization of the ENS in mouse colon was performed as previously described (42). Briefly, whole-mount preparations of the LMMP and full-thickness colon samples were fixed in 4% paraformaldehyde. For cryosections, full-thickness colon samples were dehydrated in 15% sucrose at 4°C overnight, then incubated in 15% sucrose with 7.5% gelatin at 37°C for 1 hour, snap frozen at –80°C, and sectioned at 12 μm with a Leica CM3050 S cryostat. Both whole-mount LMMP and cryostat cross-sections were permeabilized with 0.1% Triton X-100 and blocked with 10% donkey serum. Primary antibodies included anti-human HuC/D (Anna1, goat, 1:16,000; gifted by Vanda A. Lennon, Mayo Clinic, Rochester, Minnesota, USA; no commercial RRID available), rabbit anti-c-Kit antibody (1:200, Abcam, ab5506, RRID:AB_304943), rat anti-CD45 (1:500; BioLegend, 103102, RRID:AB_312967), rabbit anti-calretinin (1:200, Thermo Fisher Scientific, MA5-44580, RRID:AB_2926710), rabbit anti-neuronal nitric oxide synthase (nNOS; 1:200, Thermo Fisher Scientific, PA1-032, RRID:AB_325020), mouse anti-α-SMA (1:500, Abcam, ab5694, RRID:AB_2223021), rabbit anti-S100B (1:100, Abcam, ab52642, RRID:AB_882426), rabbit anti-smooth muscle Myosin heavy chain 11(Myh11) (1:250, Abcam, ab224804, RRID:AB_2927413), rabbit anti-PDE10A (1:500, Abcam, ab227829, RRID:AB_2927552), and rabbit anti-Parkin (1:500, Novus, NBP2-67017, RRID:AB_3353455). Secondary antibodies included donkey anti-human IgG (1:200, Alexa Fluor 647; Thermo Fisher Scientific, SA000061), donkey anti-goat IgG (1:500; Alexa Fluor 488; Thermo Fisher Scientific, A-11055, RRID:AB_2534102), goat anti-rabbit IgG (1:500, Alexa Fluor 488; Thermo Fisher Scientific, A-11034, RRID:AB_2576217), and goat anti-rat IgG (1:500; Alexa Fluor 488; Thermo Fisher Scientific, A-11006, RRID:AB_2534074). Cell nuclei were counterstained with DAPI (Vector Labs) and mounted with Aqua-Poly/Mount (Fisher Scientific Polysciences Inc.). Images were obtained with a Nikon A1R laser scanning confocal microscope or a Keyence BZX-700 All-In-One microscope.

### Quantitative analyses of immunohistochemical, histological, and morphological data

Myenteric neurons immunoreactive for Hu, nNOS, and calretinin were quantified in whole mounts within a 2 mm^2^ area by randomly capturing 8 original magnification, 20×, images per preparation. Leukocyte infiltration was assessed by manually counting CD45^+^ cells in full-thickness colonic sections (1.5 mm^2^) encompassing mucosa and muscle. ICC labeling in distal colon cross sections was analyzed from 4 original magnification, 20×, images per preparation (1 mm^2^). All images were captured under identical exposure settings and calibrated to a standardized fluorescence baseline.

Images were processed in ImageJ (NIH), converted from RGB to 8-bit grayscale, thresholded, binarized, and analyzed for percentage area of CD45 and ICC immunoreactivity. Muscle thickness was quantified from H&E-stained sections; however, smooth muscle cell number and size were not separately assessed, so the cause of thinning (cell loss vs. atrophy) remains undetermined.

### Simultaneous measurement of CMMC video and intraluminal pressure

Organ baths were maintained in Krebs solution at 36.5°C ± 0.5°C and bubbled with carbogen (95% O_2_/5% CO_2_). After dissection, colons were flushed, cannulated at both ends, and mounted horizontally in the bath. The proximal cannula was connected to a Krebs reservoir (15 mL) positioned to maintain a constant 2 cm H_2_O intraluminal pressure. The distal cannula was connected to a pressure transducer (CWE Inc.) with the outflow tube held vertically to sustain equal backpressure.

Intraluminal pressure was digitized and recorded using a PowerLab 16/35 system with LabChart Pro v8.1.16 (ADInstruments). Simultaneously, a video camera (Logitech Quickcam Pro) positioned approximately 8 cm above the organ bath captured three 10-minute recordings. Videos were processed into spatiotemporal maps (diameter vs. time) using the Gastrointestinal Motility Monitoring (GIMM) system (Med-Associates) and temporally aligned with pressure traces for correlation analysis.

### Analysis of CMMC parameters

CMMCs were defined as coordinated propagating contractions traveling in the oral-to-anal direction that spanned at least 50% of the colon length. Frequency, velocity, and duration of CMMCs were analyzed using the GIMM processor plugin (ImageJ) (43). Diameter measurements in the spatiotemporal maps were derived from edge-detection analysis of the video recordings; no separate manual measurement of diameter was performed.

### Measurement of total GI transit time

Total GI transit was measured after oral gavage of 150 μL of 6% (w/v) carmine red (Sigma-Aldrich) in 0.5% (w/v) methylcellulose using a 21-gauge round-tip feeding needle. Food was withheld for 2 hours before gavage. Mice were housed individually with free access to food and water, and the time from gavage to expulsion of the first red-stained fecal pellet was recorded as total transit time. Fecal output was checked every 10 minutes, after which mice were returned to standard housing.

### Rectal bead expulsion measurement

Distal colonic motility was assessed by rectal bead expulsion. Mice were food-deprived for 1 hour and lightly anesthetized with isoflurane (Covetrus, 11695-6777-2). A 3 mm glass bead (Sigma-Aldrich, 1040150500) was gently inserted 1 cm into the distal colon using a lubricated applicator. After recovery (indicated by spontaneous righting), mice were returned to their cages, and the time to bead expulsion was recorded. Each animal underwent 3 trials, and the mean expulsion time was calculated. Mice were then provided food and water and returned to standard housing.

### Measurement of gastric emptying

Gastric emptying (solid and liquid) was assessed as described previously (44). Mice received intragastric gavage of 0.3 mL barium sulfate suspension (X-OPAQUE-HD, 2.5 g/mL) containing 10 steel beads (0.81–0.9 mm; Bal-tec). Radiographs were taken 90 minutes after gavage using a portable x-ray unit (50 kV, 1.2 mAs; ScanX14, ALLPRO Imaging). Images were analyzed in ImageJ. Solid gastric emptying was expressed as the percentage of beads expelled from the stomach, and liquid emptying was determined by the ratio of barium signal integrated density outside the stomach to the total GI tract signal.

### Measurement of smooth muscle activity using EFS

Organ bath experiments were performed as described previously (45). Freshly excised distal colon (used for all contractility and pharmacological assays), along with mid and proximal colon (for regional EFS studies; Supplemental Figure 2G) and ileum, were placed in oxygenated Krebs solution. Tissues were cut into 5 mm rings and mounted on force transducers in a muscle strip myograph bath (model 820 MS; Danish Myo Technology) at 37°C. Rings were stretched to 0.5 g resting tension and equilibrated for 30–45 minutes, with Krebs refreshed every 20 minutes.

EFS was applied using 40–50 V, 15-second pulse trains (300 μs, 5 Hz) via a CS4^+^ stimulator and Myo Pulse software (DMT). Contractile force was recorded using a Power Lab 16/35 system and analyzed with Lab Chart Pro v8.1.16 (ADInstruments).

For pharmacological assays Ach (0.01–100 μM; Sigma-Aldrich) was used to assess maximal contraction, TTX (0.5 μM; Alomone Labs) to block neurogenic input, and KCl (60 mM) to confirm tissue viability. Relaxation responses were measured using sodium nitroprusside (0.01–100 μM; Sigma-Aldrich). Ileal contractility was assessed using identical protocols for regional comparison.

### Data acquisition and analysis of organ bath studies

Baseline values were obtained from a 60-second segment recorded 1 minute before EFS or compound addition. For EFS, ACh, and KCl experiments, the maximal contractile force within 5 minutes after stimulation was measured as the absolute change (Δg) from baseline. In tissues with spontaneous rhythmic contractions (e.g., ACTA2 mutants), the highest peak during this window was used. EFS was repeated 3 times at 5-minute intervals, and the mean of the 3 responses was taken as the final value.

Spontaneous activity was quantified as AUC (g × s) from 60-second baseline recordings. To assess neuronal blockade, AUC was measured before and after TTX addition. Unless otherwise noted (e.g., regional assays in Supplemental Figure 2G), all recordings were from distal colon.

For CMMC recordings, luminal pressure was defined as the maximum intraluminal pressure (mmHg) within a 10-minute kymograph period using a pressure transducer. In controls, this corresponded to a propagating CMMC; in ACTA2 mutants, the highest transient pressure observed was used.

### snRNA-seq

Nuclei were isolated from frozen tissue using the 10x Genomics Chromium Nuclei Isolation Kit for tissues (PN-1000494) following the manufacturer’s protocol for solid samples. All steps were performed on ice or at 4°C unless otherwise specified.

Colons were collected and pinned in a silicone-lined dish for removal of the mucosa and submucosa. The muscularis propria was minced into 2–5 mm fragments, transferred to pre-chilled dissociation tubes, lysed with 200 μL nuclei isolation buffer, and mechanically dissociated with a sterile pestle. After adding 300 μL lysis buffer, samples were pipette-mixed and incubated on ice for 10 minutes. Homogenates were filtered through a pre-chilled nuclei isolation column, and nuclei were collected by centrifugation (16,000*g*, 20 seconds, 4°C).

The crude nuclei were pelleted (500*g*, 3 minutes, 4°C), resuspended in 500 μL debris removal buffer, mixed, and centrifuged again (700*g*, 10 minutes, 4°C). Pellets were washed twice in 1 mL wash and resuspension buffer (500*g*, 5 minutes, 4°C each), then resuspended in 50 μL wash and resuspension buffer. Nuclei suspensions were gently mixed, counted by DAPI staining, adjusted to the recommended concentration for 10x Genomics Chromium Single Cell Gene Expression assays, kept on ice, and processed immediately. snRNA-seq data were obtained from 1 ACTA2 mutant mouse and 1 littermate control of each sex using the Chromium Single Cell 3’ Reagent Kit (v3.1). Cell Ranger filtered output files were read into R using the Read10X function of *Seurat*. Initial quality control steps included filtering low-quality cells and removing confounding gene sets. Genes linked to immediate early response, sex-linked genes, heat shock proteins, and ribosomal proteins were removed from the dataset. The cells were further filtered based on the number of detected features and mitochondrial gene content. Cells with fewer than 200 or more than 3,000 features, less than 500 unique molecular identifier counts, and cells with more than 10% mitochondrial gene content were excluded from analysis.

The data were normalized using the Seurat function NormalizeData to account for sequencing depth. The top 2,000 highly variable features were identified using FindVariableFeatures, and the data were scaled to ensure comparability. PCA was conducted using RunPCA to reduce dimensionality, and the most significant principal components (PCs) were selected based on the explained variance. The number of PCs retained was determined by evaluating the cumulative explained variance. PCs were retained until the cumulative explained variance exceeded approximately 90%, while avoiding inclusion of PCs with minimal individual contributions (<5%) unless required to reach this threshold. Using this approach, at least the minimum number of PCs was considered for downstream analyses, and in the final analysis, 21 PCs were used. Nearest neighbors were identified using FindNeighbors, clusters were determined with FindClusters at a resolution of 0.1, and clusters were visualized in 2 dimensions using UMAP (RunUMAP). Doublet detection was performed using the scds package, applying both bcds and cxds methods. A hybrid method (cxds_bcds_hybrid) was used when both scores were available. Detected doublets were excluded from subsequent analyses. After clustering, small clusters containing fewer than 50 nuclei were removed from the dataset prior to downstream analyses as clusters with very few cells are unlikely to represent biologically meaningful data between genotypes. The final numbers of nuclei analyzed per cell type are shown in Supplemental Table 7. Module scores for contraction and proliferation were assessed as described by Kwartler et al. (25) after reciprocal principal component analysis–based integration between the datasets.

Single-cell DEGs were calculated using the Wilcoxon rank-sum test in Seurat’s FindMarkers() function. Comparisons were made between mutant and control cells within each cluster. Only genes expressed in at least 20% of cells in either group and with log_2_ fold-change greater than 0.5 were retained, and DEGs were filtered for adjusted *P* value less than 0.05. Pseudo-bulk DEGs were calculated by aggregating raw counts across cells from the same cluster, genotype, and replicate. Differential expression was performed using DESeq2 with the design formula ~ genotype, modeling gene expression as a function of genotype. Wald tests were used to identify DEGs, and genes with adjusted *P* value less than 0.05 and absolute log_2_ fold-change greater than 0.5 were reported. Nonsignificant or missing values were excluded. Overrepresentation analysis was conducted using the *msigdbr* mouse dataset for GO biological processes using the enricher function of the clusterProfiler package. Gene sets with a log_10_ adjusted *P* value greater than 3 were retained. Enrichment results were merged across clusters and filtered to retain only the most significant pathways (adjusted *P* value < 0.05). Cluster results were visualized using a heatmap to depict log_2_FC values for the top enriched terms per cluster, with hierarchical clustering applied for both genes and GO term using the ComplexHeatmap package.

### Flow cytometric immune profiling

Full-thickness colonic tissue was cleared of mesenteric fat, opened along the mesenteric border, and washed in ice-cold PBS. Samples were minced and digested in collagenase type XI (1 mg/mL; Sigma-Aldrich, C7657) and dispase (0.4 U/mL; STEMCELL Technologies) in DMEM (Gibco) for 60 minutes at 37°C with agitation. Digests were triturated, filtered twice through 70 μm strainers (Corning, 352350), and centrifuged (350*g*, 5 minutes, 4°C) to obtain single-cell suspensions.

Cells were blocked with anti-CD16/CD32 (BioLegend, 101301) in PBS plus 2% FBS plus 1 mM EDTA for 5 minutes at room temperature, then stained for 30 minutes at 4°C with fluorophore-conjugated antibodies: CD3 APC (Invitrogen, 17-0032-82), CD4 PE/Cy7 (BioLegend, 100421), CD8a FITC (BioLegend, 100705), CD11b APC (BioLegend, 101211), CD45 BV510 (BioLegend, 103137), F4/80 AF488 (Invitrogen, MF48020), Ly6C BV650 (BioLegend, 128049), and Ly6G PerCP/Cy5.5 (BioLegend, 127615). Dead cells were excluded using Fixable Viability Dye eFluor 780 (Invitrogen, 65-0865-14).

Data were acquired on a FACSAria III cell sorter (BD Biosciences) with FACSDiva software and analyzed in FlowJo (v10.10).

### Statistics

Grouped data are expressed as mean ± SEM. Statistical analysis was conducted with GraphPad Prism (version 9). “Peak effect” refers to the maximum contractile or relaxation response observed in functional assays. For comparisons between 2 groups with normally distributed data and equal variances, an unpaired 2-tailed *t* test was used. If normality assumptions were not met, the nonparametric Mann-Whitney *U* test was applied. For concentration-response experiments (ACh and sodium nitroprusside), a 2-way repeated-measures ANOVA was used to assess genotype and dose interactions, followed by Bonferroni’s post hoc test for multiple comparisons. For all comparisons, *P* less than 0.05 was considered statistically significant.

### Study approval

#### Study participants.

The review of clinical data from patients with ACTA2 R179 variants enrolled in the YGSA study at Massachusetts General Hospital was approved by the Mass General Brigham IRB (protocol 2018P002134). Written informed consent was obtained from adult participants or legal guardians; assent was obtained from minors when appropriate.

#### Animal studies.

All animal procedures were approved by the IACUC at Massachusetts General Hospital (protocols 2018N000067 and 2013N000115).

### Data availability

The snRNA-seq dataset generated in this study has been deposited in NCBI’s Gene Expression Omnibus under accession number GSE305376, which is publicly available. All analysis code is available on GitHub (https://gist.github.com/rhianstavely/f90fe854273f46a0d3f2f3641dce14c5).

All supporting data values associated with the main manuscript and supplemental figures, including individual data points underlying graphs and summary values, are provided in a separate Supporting Data Values Excel file. Each figure panel is presented on a dedicated tab with clear labeling.

## Author contributions

AAR, MEL, PLM, and AMG designed the research studies. AAR, RS, and LCO conducted experiments, analyzed data, and interpreted results of experiments. EDC and DT collected clinical data. RH, SD, CYH, and KO provided resources and technical support. AAR, RS, and AMG drafted the manuscript. AAR, AJB, MEL, PLM, and AMG edited and revised the manuscript. All authors approved the final version of manuscript.

## Funding support

This work is the result of NIH funding, in whole or in part, and is subject to the NIH Public Access Policy. Through acceptance of this federal funding, the NIH has been given a right to make the work publicly available in PubMed Central.

- NIH 5R01NS125353 (to PLM and MEL).
- AAR by an Eleanor and Miles Shore Faculty Development Award and the American Neurogastroenterology and Motility Society Discovery Grants Program.

## Supplementary Material

Supplemental data

Supplemental table 3

Supplemental table 4

Supplemental table 6

Supplemental video 1

Supporting data values

## Version 1 01/06/2026

In-Press Preview

## Version 2 02/23/2026

Electronic publication
