# Supplementary material for: Structural and functional gastrointestinal abnormalities in ACTA2 R179H mice modeling multisystemic smooth muscle dysfunction syndrome
Source: JCI Insight. 2026 Jan 6;11(4):e190469. doi: 10.1172/jci.insight.190469 (PMC12956000; doi:10.1172/jci.insight.190469)
Supplement: Supplemental data [file jciinsight-11-190469-s237.pdf]

Revised Supplementary Figure 1

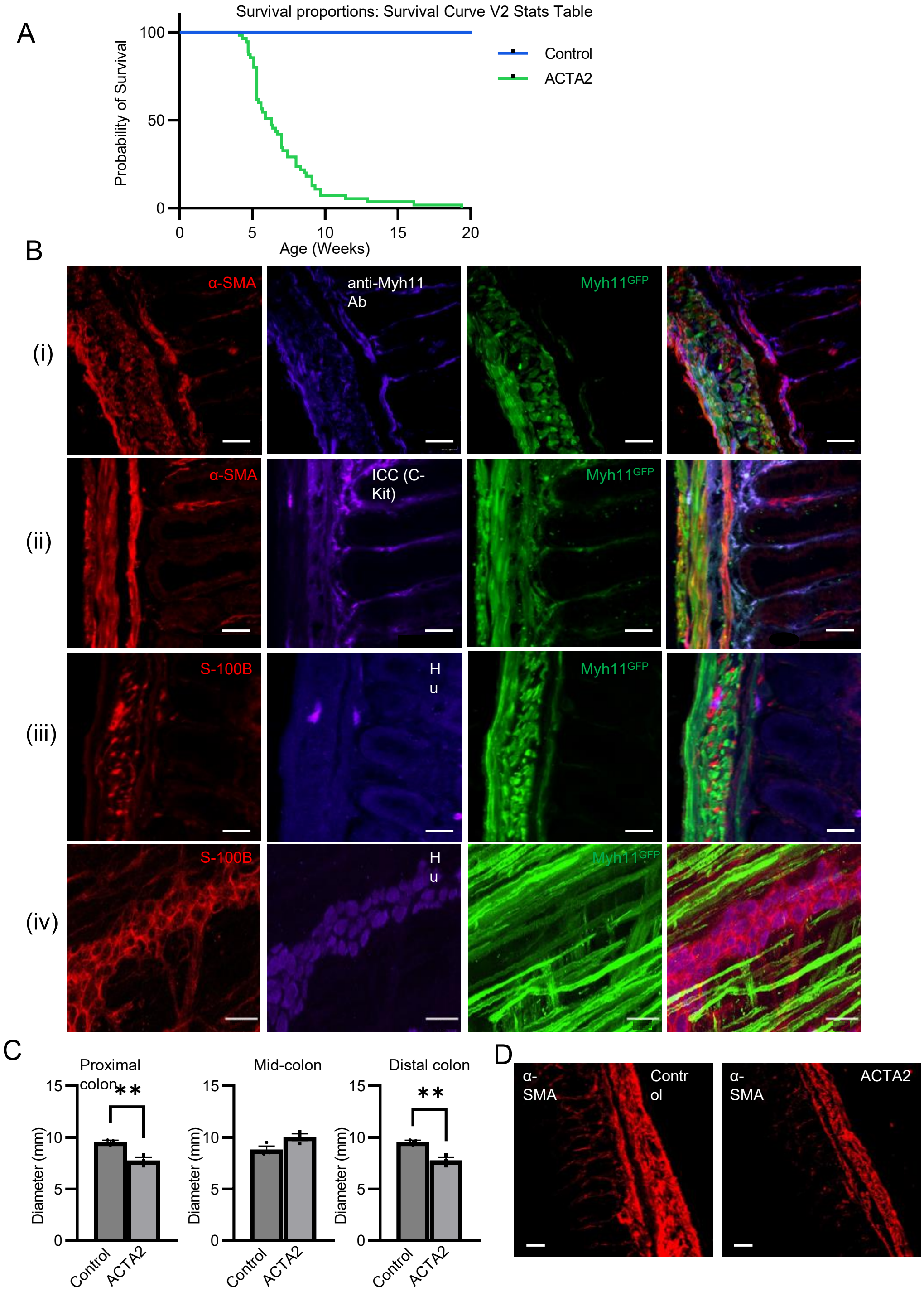

Supplementary Figure 1. Reduced survival, smooth muscle–specific recombination and structural remodeling of the colon in ACTA2 mutant mice. (A) Kaplan–Meier survival analysis showing markedly reduced lifespan in ACTA2 mutant mice compared with controls. B) Validation of Myh11-Cre specificity. Immunofluorescence labeling of EGFP (green) with α-SMA and MYH11 (red) showed complete overlap in the muscularis propria, confirming smooth muscle–specific recombination (i). No co-localization of EGFP was observed with interstitial cells of Cajal (c-Kit) (ii), or with glial cells (S-100B) and enteric neurons (Hu) in cryostat sections (iii) or in whole-mount longitudinal muscle-myenteric plexus preparations (iv), indicating absence of off-target Cre activity. (C) Regional measurements of colonic diameter in maximally stretched and pinned colons. (D) α-SMA (ACTA2) immunofluorescence staining of distal colon. Scale bar = 50 μm. Data are presented as mean ± SEM. Statistical comparisons were made using unpaired t-tests, as detailed in Methods. **$p < 0.01$.

Supplementary Figure 2

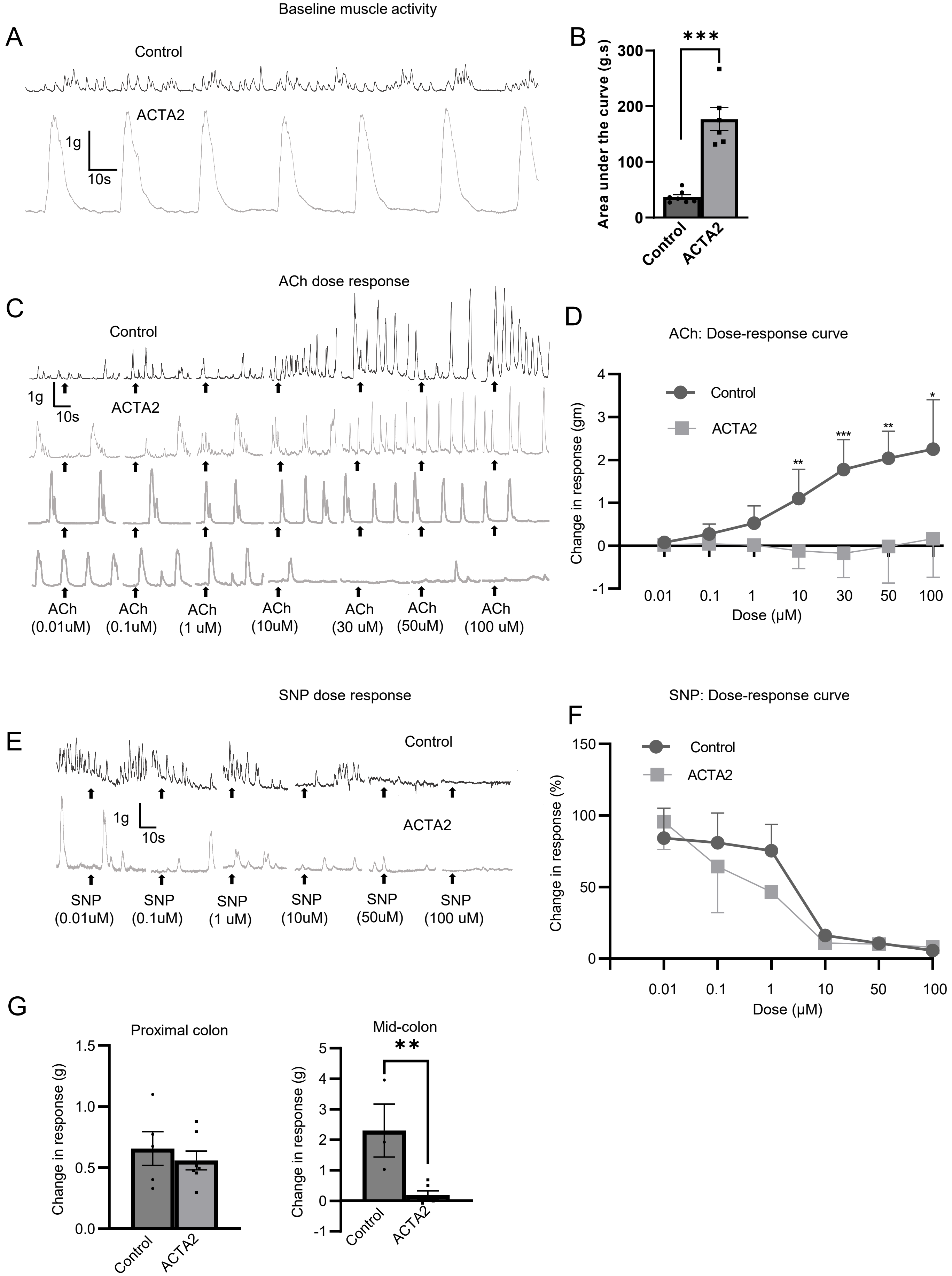

Supplementary Figure 2. Spontaneous activity, cholinergic responses, SNP-induced relaxation, and regional variation in ACTA2 mutant colon. (A) Representative traces of spontaneous activity in distal colon. ACTA2 mutants showed rhythmic, high-amplitude contractions absent in controls. (B) Quantification of baseline activity (AUC) confirmed significantly elevated spontaneous contractions in ACTA2 mutants. (C) Representative traces from acetylcholine (ACh; 0.01–100 μM) dose–response experiments. Controls displayed robust, graded contractions, while ACTA2 mutants showed variable responses: small increases (i), no response (ii), or paradoxical suppression at high doses (iii). (D) Group dose–response curve for ACh. Controls showed a steep increase in force; ACTA2 mutants showed no significant response. (E) Representative traces of sodium nitroprusside (SNP)–induced relaxation. Both groups showed dose-dependent decreases in tone. (F) Group dose–response curve for SNP, demonstrating comparable relaxation in controls and mutants. (G) EFS-induced contractile responses in proximal and mid-colon. Controls exhibited robust contractions, while ACTA2 mutants showed significant reduction in the mid-colon but preserved responses in the proximal colon. Data are presented as mean ± SEM. Statistical comparisons were made using unpaired t-tests or two-way ANOVA with Bonferroni correction, as detailed in Methods. *$p < 0.05$, **$p < 0.01$, ***$p < 0.001$.*

Supplementary Figure 3

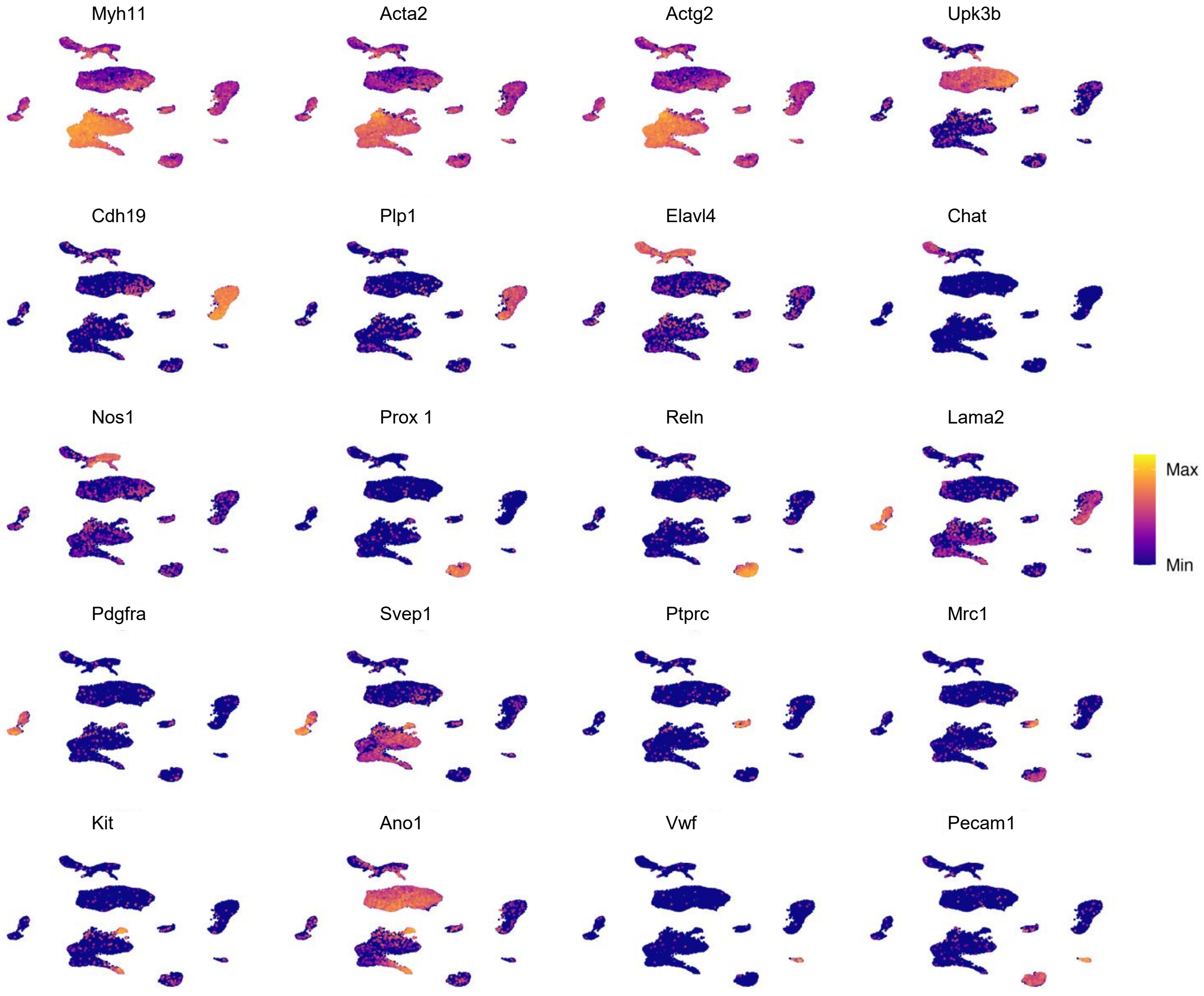

Supplementary Figure 3. Additional feature plots for refined cell type annotation. Feature plots showing expression of additional markers used to refine cluster annotation. Actg2 distinguished visceral from vascular smooth muscle cells. Elavl4 confirmed neuronal cluster identity alongside Chat and Nos1. Svep1 and Lama2 supported the classification of Pdgfra+ clusters as PDGFRA+ SIP cells rather than generic fibroblasts. Mrc1 expression identified macrophages within the Ptprc+ immune cell population.

Supplementary Figure 4

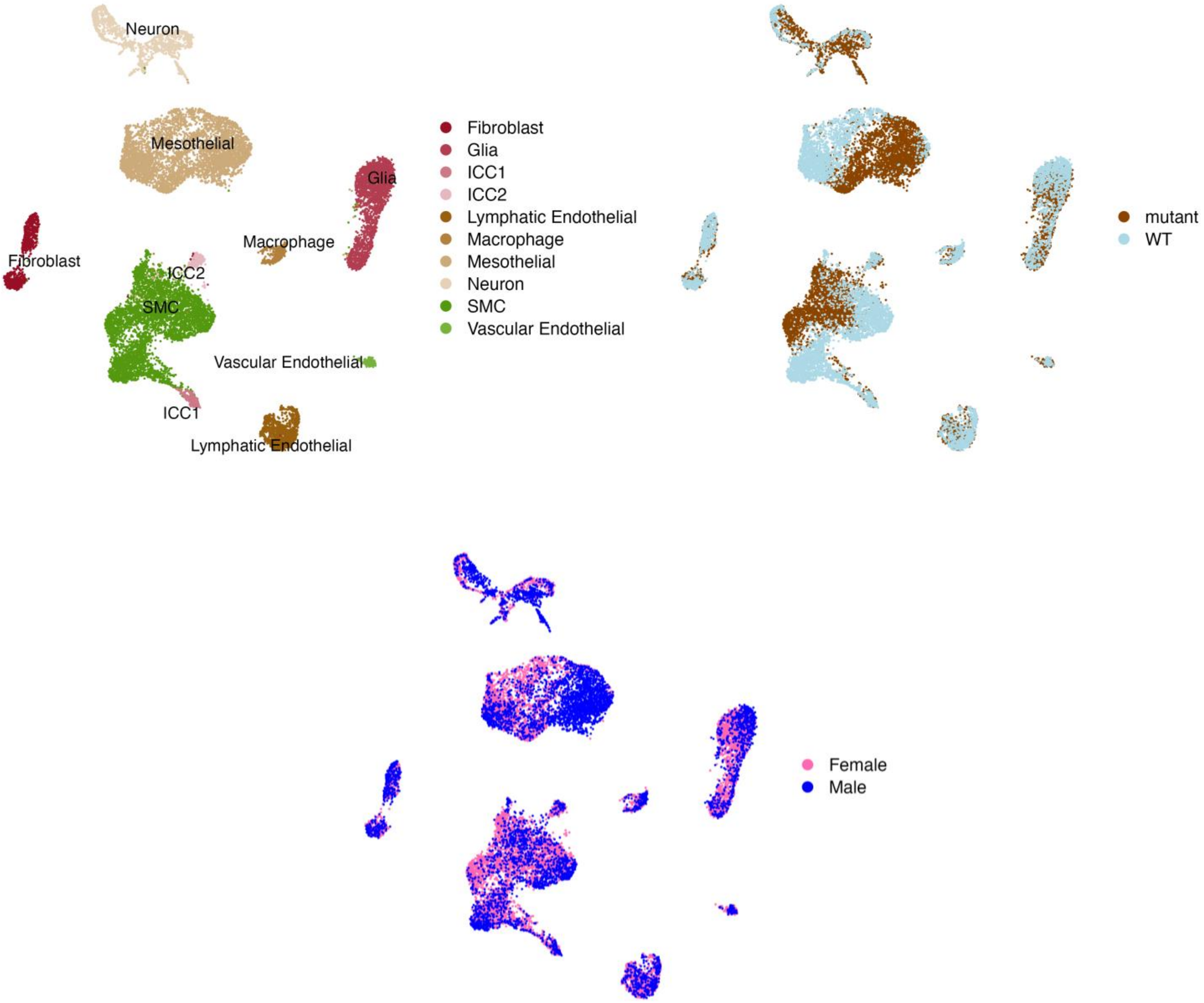

Supplementary Figure 4. UMAP projection of snRNA-seq dataset colored by cell type annotations (top left) genotype (top right) and sex (bottom). Both male and female mice contributed to all identified clusters, and no distinct segregation by sex was observed.

Supplementary Figure 5

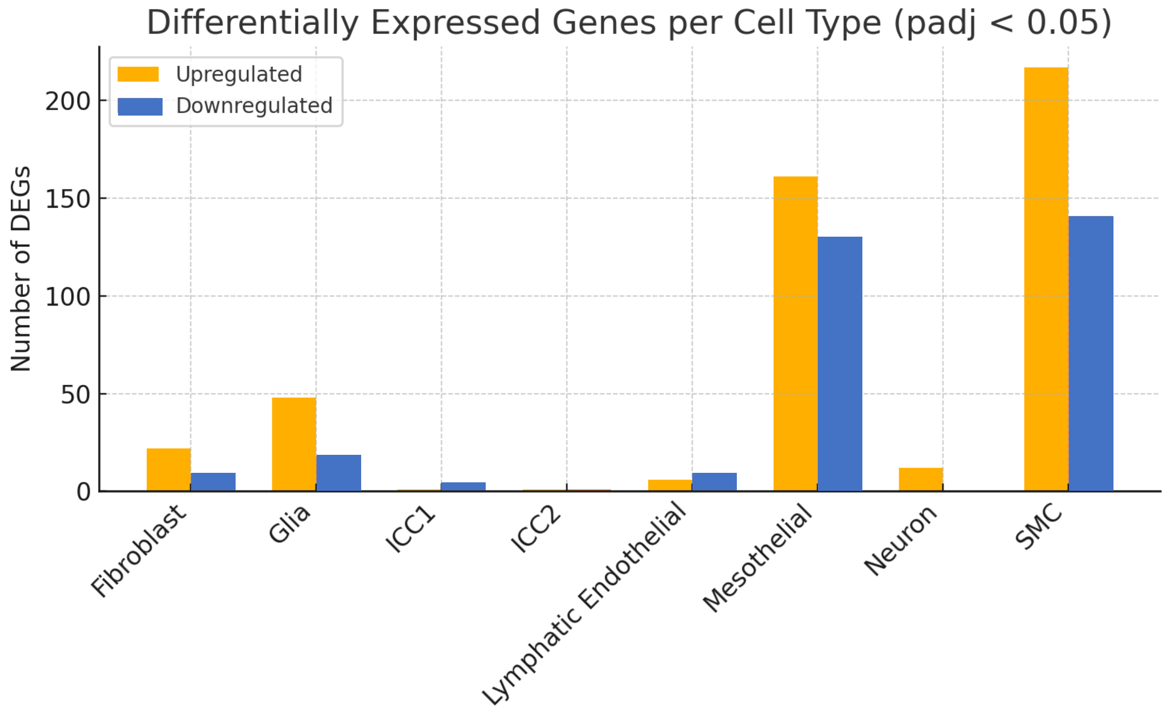

Supplementary Figure 5. Differentially expressed genes (DEGs) in each colonic muscularis externa cell type in ACTA2 mutant versus control mice. Bar chart shows the number of upregulated (yellow) and downregulated (blue) genes per annotated cluster, based on pseudobulk analysis with adjusted p-value < 0.05. While smooth muscle cells exhibited the largest number of DEGs, mesothelial cells, glia, fibroblasts, and neurons also displayed measurable transcriptional changes. ICC subtypes exhibited few DEGs.

Supplementary Figure 6

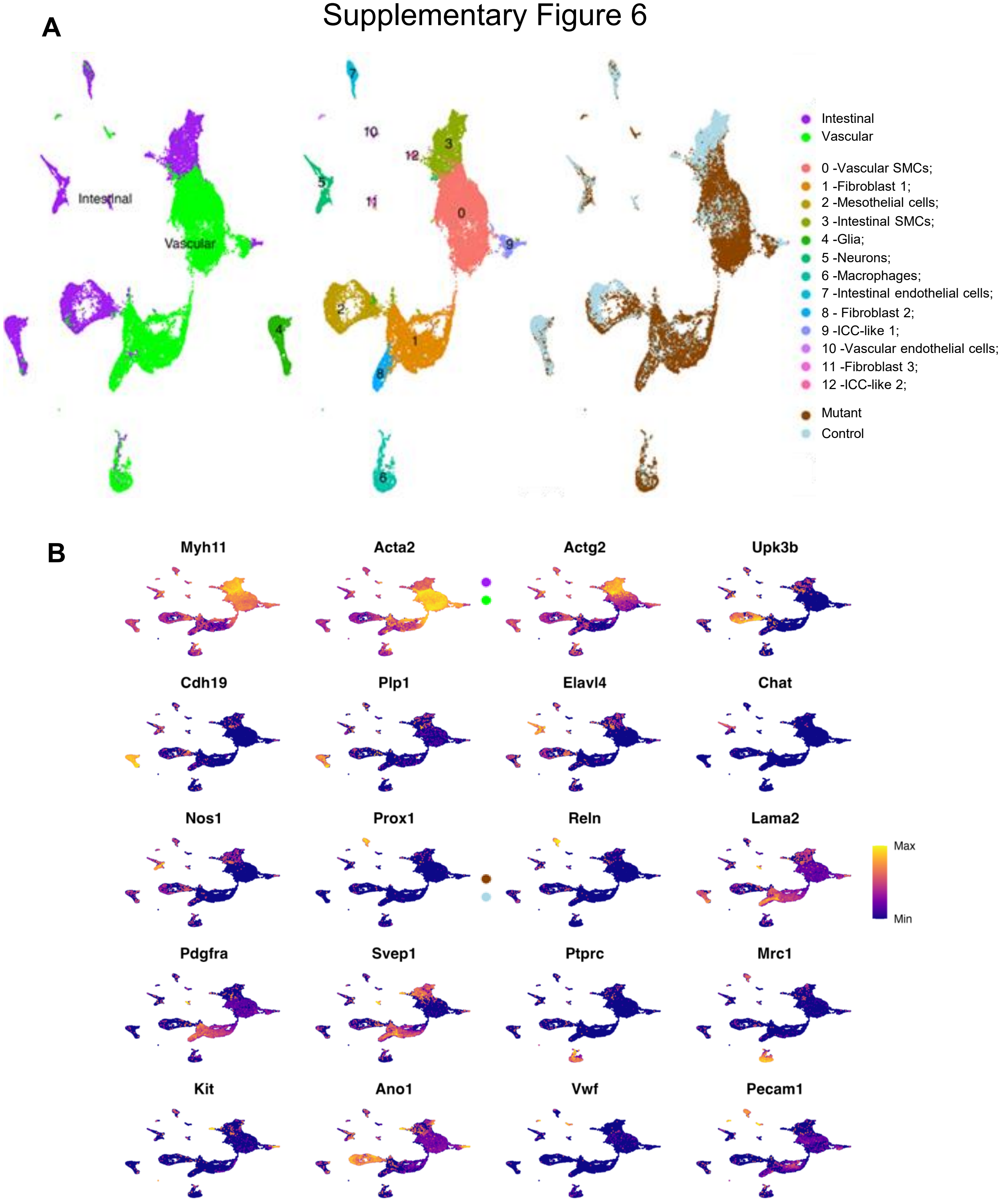

Supplementary Figure 6. Integrated analysis of intestinal and vascular smooth muscle datasets reveals conserved and tissue-specific effects of ACTA2 mutations.
(A) UMAP of integrated intestinal smooth muscle (purple) and vascular smooth muscle (green; Kwartler et al., 2023) single-cell datasets, colored by tissue of origin (left), transcriptional cluster (middle), and genotype (right). ACTA2-mutant cells (brown) are enriched in shared clusters spanning both tissues. (B) Feature plots showing expression of canonical smooth muscle contractile genes (**Myh11**, **Acta2**, **Actg2**) and markers for other lineages (urothelium, Schwann cells, neurons, lymphatic endothelium, fibroblast-like cells, immune cells, interstitial cells of Cajal, and vascular endothelium), confirming smooth muscle identity of major clusters.

Supplementary Figure 7

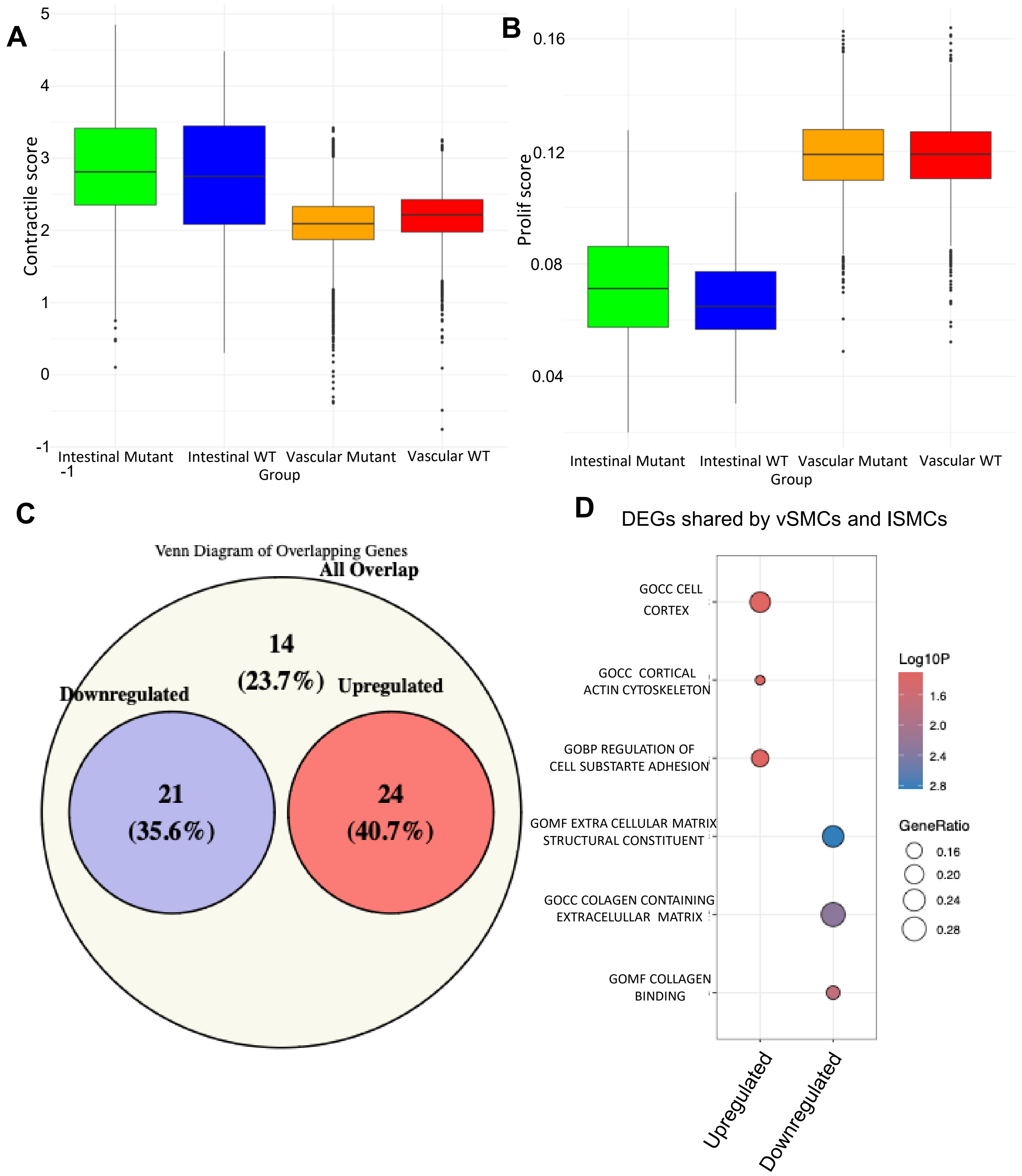

Supplementary Figure 7. Comparison between vascular smooth muscle cells (VSMCs) and intestinal smooth muscle cells (ISMCs) related to Supplementary Figure 6. (A) Contractile program module scores in the major smooth muscle clusters for intestinal and vascular smooth muscle cells by genotype. ACTA2-mutant vascular smooth muscle cells show significantly reduced contractile scores compared to wild type, whereas intestinal smooth muscle cells have higher absolute scores and no genotype-dependent decrease. (B) Proliferative program module scores. ACTA2-mutant vascular smooth muscle cells show modestly increased proliferative scores relative to wild type, while intestinal smooth muscle cells display lower scores overall with no marked genotype-related difference. (C) Venn diagram showing the overlap in DEGs caused by ACTA2 mutations in VSMCs and ISMCs. (D) ORA analysis of gene ontologies enriched in the DEGs shared by shared VSMCs and ISMCs as above.

Supplementary Figure 8

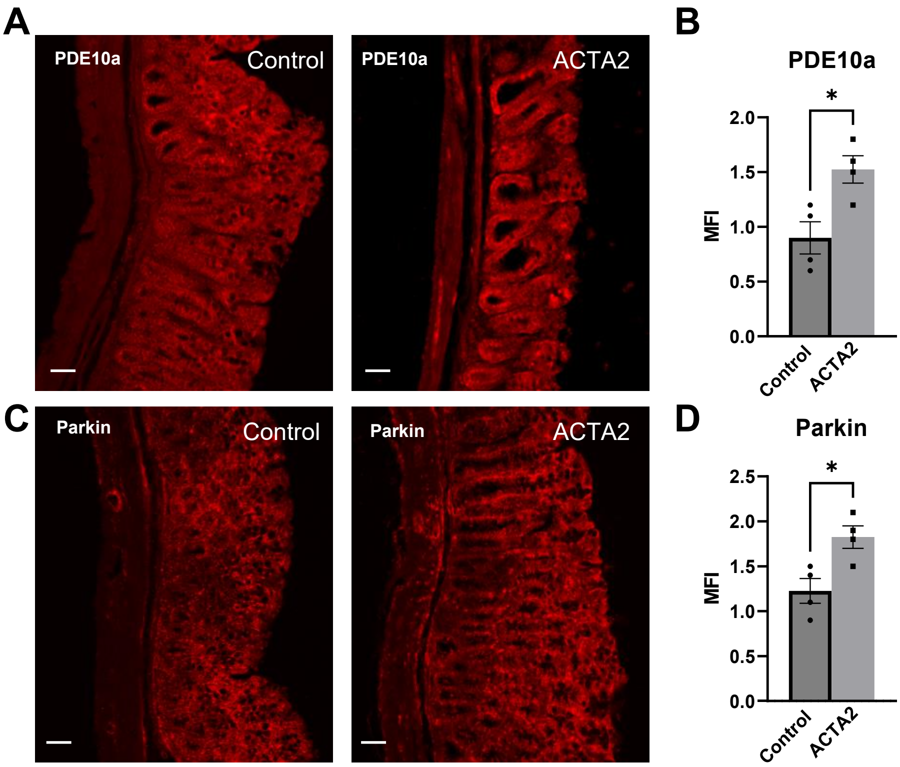

Supplementary Figure 8. Validation of snRNA-seq–identified genes Pde10a and Prkn in ACTA2 mutant distal colon. (A, B) Representative immunofluorescence images and quantification of PDE10A mean fluorescence intensity (MFI) in the distal colonic muscularis externa of control and ACTA2 mutant mice. PDE10A expression was significantly elevated in mutants. (C, D) Representative images and quantification of Parkin immunoreactivity, which was also significantly increased in ACTA2 mutants compared with controls. Scale bar = 50 µm. Data are presented as mean ± SEM. Statistical comparisons were made using unpaired t-tests, as detailed in Methods. *$p < 0.05$.

Supplementary Figure 9

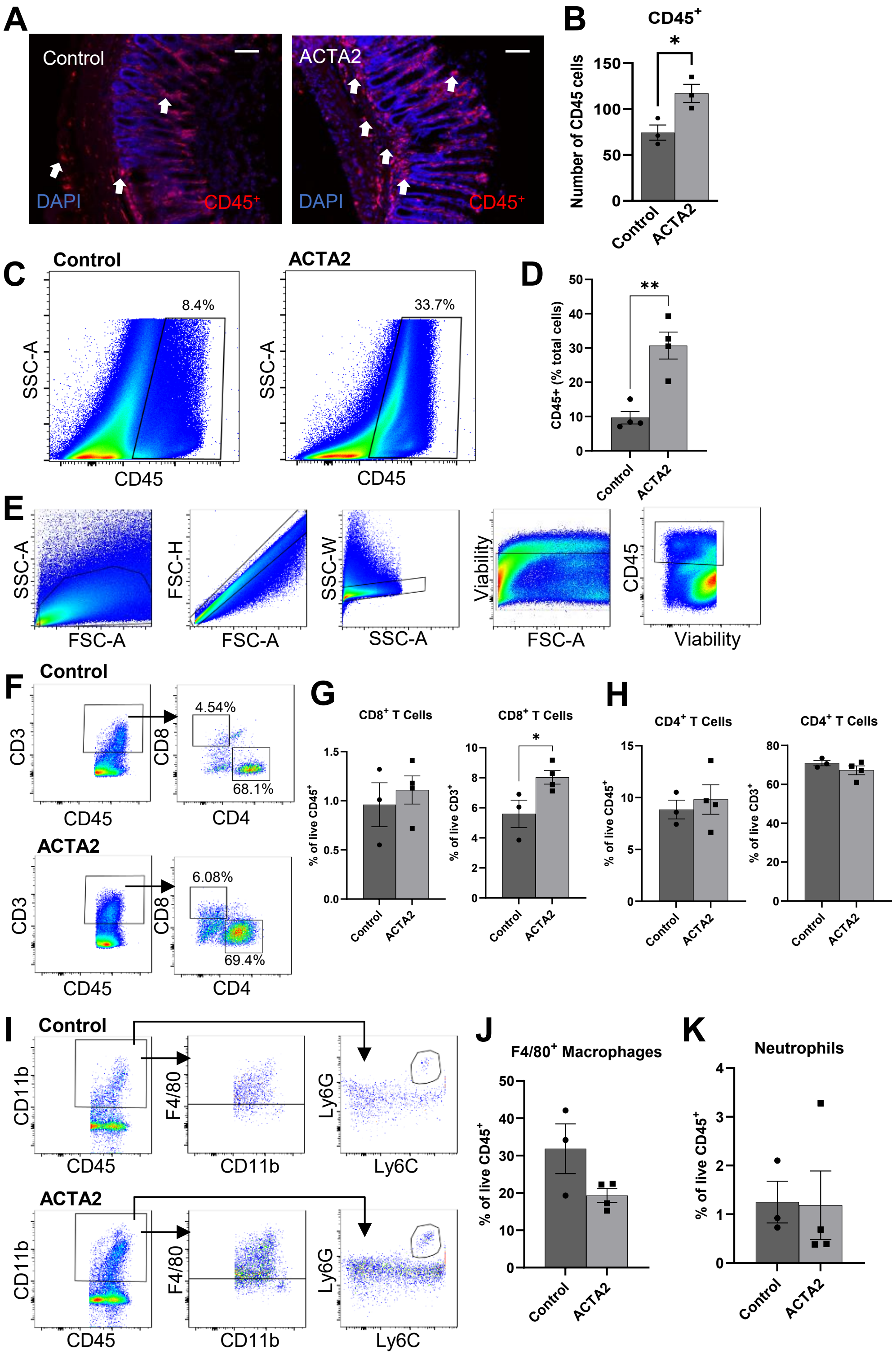

Supplementary Figure 9. **ACTA2 mutation is associated with elevated immune cell infiltration in the colon.** (A) Representative immunofluorescence images of full-thickness colon stained for CD45 (red) and counterstained with DAPI (blue) to define tissue structure. White arrows indicate CD45+ cells. B) Quantification of $CD45^+$ immune cells in the colon from immunofluorescence images of full-thickness colonic cross sections (total analyzed area = 1.5 mm^2) encompassing both mucosal and muscular layers. Data represent manual cell counts, not flow cytometry, and complement the flow cytometric analyses shown in subsequent panels. (C) Representative flow-cytometric plots of colonic $CD45^+$ cells in in full-thickness colon samples from ACTA2 mice and controls. Numbers indicate percentage of cells inside the gate. (**D**) Quantification of CD45+ cells in full-thickness colon samples from control (n=4) and ACTA2 (n=4) mutant mice (as a percentage of all cells). Data are mean ± SEM, unpaired *t* test, $^{**}p < 0.01$. (**E**) Gating strategy to identify single, live CD45+ cells prior to further myeloid and lymphoid subpopulation analysis shown below (**F**) Flow-cytometric plots of colonic T cell populations (gated from live $CD45^+$ cells) in ACTA2 mice and controls. Numbers indicate percentage of cells inside the gate. (**G**) Quantification of colonic $CD8^+$ T cells in ACTA2 mice and controls (n = 3-4 per group), relative to live $CD45^+$ immune cells and live $CD3^+$ T cells. $^*p < 0.05$. . (**H**) Quantification of colonic $CD4^+$ T cells in ACTA2 mice and controls (n = 3-4 per group), relative to live $CD45^+$ immune cells and live $CD3^+$ T cells. (**I**) Representative flow-cytometric plots of myeloid subpopulations (from CD45+, live single cells), first gated on CD11b followed by definition of F4/80+ macrophages and $Ly6C^{hi}$ Ly6G+ neutrophils. Quantification of colonic macrophages (**J**) and neutrophils (**K**) in ACTA2 mice and controls (n = 3-4 per group), relative to live $CD45^+$ immune cells. Scale bar = 50 µm. Data are presented as mean ± SEM. Statistical comparisons were made using unpaired t-tests, as detailed in Methods. $^*p < 0.05$, $^{**}p < 0.01$.

Supplementary Table 1. Clinical data supporting gut dysmotility in MSMDS

Patient Number	Age (years)	Sex	Genetic mutation	Use of Laxatives or Enemas	Need for Enteral Nutrition	Use of Prokinetics
1	0.5	M	R179H	No	Yes	No
2	1	F	R179H	Yes	No	Yes
3	2	F	R179H	Yes	Yes	No
4	2	F	R179H	No	Yes	No
5	2	F	R179H	Yes	Yes	No
6	5	M	R179H	Yes	No	Yes
7	6	F	R179H	Yes	No	No
8	7	M	R179H	Yes	Yes	Yes
9	7	F	R179H	No	No	Yes
10	8	M	R179H	Yes	Yes	No
11	9	M	R179H	Yes	No	No
12	9	F	R179C	Yes	No	No
13	9	F	R179H	Yes	Yes	No
14	11	F	R179H	Yes	No	No
15	13	F	R179H	Yes	Yes	No
16	13	M	R179C	Yes	No	No
17	14	F	R179H	Yes	No	Yes
18	15	F	R179H	Yes	Yes	No
19	16	M	R179H	No	No	No
20	16	F	R179C	Yes	No	No
21	17	F	R179H	No	No	No
22	20	F	R179H	Yes	Yes	Yes
23	22	M	R179H	Yes	Yes	No
24	26	M	R179H	No	Yes	No

Supplementary Table 2. Caregiver survey data for gastrointestinal symptoms in patients with ACTA2 R179 variants

**Metric**	**Most Common Baseline Response**	**% (out of 14 respondents)**
Bowel Movement Frequency	More than once a day	53.80%
Difficulty or Pain Opening Bowels	Rarely	38.50%
Feeling of Incomplete Evacuation	Sometimes	46.20%
Pain in Abdomen	Sometimes	53.80%
Time to Open Bowels	5 -10 minutes	46.20%
Duration of Constipation Issues	5 -10 years	46.20%
Need for Assistance	No assistance (i.e. no laxatives or digital assistance needed)	53.80%
Average Stool Type (Bristol Stool Chart)	Type 6	30.80%

Supplementary Table 5. Differentially expressed genes per cell type in ACTA2 mutant versus control colonic muscularis propria

Cell Type	Upregulated	Downregulated	Total DEGs
Fibroblast	22	9	31
Glia	48	18	66
ICC1	1	4	5
ICC2	1	1	2
Lymphatic Endothelial	6	9	15
Mesothelial	161	130	291
Neuron	12	0	12
SMC	217	141	358

Supplementary Table 5. Number of upregulated, downregulated, and total differentially expressed genes (DEGs) per cell type in ACTA2 mutant versus control mice, based on pseudobulk single-nuclei RNA-seq analysis (adjusted p-value < 0.05).

Supplementary Table 7. Nuclei count per cell type in control and ACTA2 mutant colonic muscularis propria.

	genotype	orig.ident	Fibroblast	Glia	ICC1	ICC2	Lymphatic Endothelial	Macrophage	Mesothelial	Neuron	SMC	Vascular Endothelial	Total Nuclei
1	control	RSb24	300	631	167	83	367	156	1345	293	1747	33	5122
2	mutant	RSb25	236	977	62	100	381	106	701	427	1672	87	4749
3	control	RSb26	255	388	53	28	225	112	467	114	998	40	2680
4	mutant	RSb27	108	263	19	21	124	43	2035	663	496	15	3787

Number of nuclei assigned to each annotated cell type after final quality control and clustering.
